# Sparse vertex discriminant analysis: Variable selection for biomedical classification applications

**DOI:** 10.1016/j.csda.2025.108125

**Published:** 2025-01-07

**Authors:** Alfonso Landeros, Seyoon Ko, Jack Z. Chang, Tong Tong Wu, Kenneth Lange

**Affiliations:** a Department of Statistics, University of California, Riverside, CA, 92521-0001, United States of America; b Departments of Mathematics and Biostatistics, University of California, Los Angeles, CA, 90095-1554, United States of America; c Translational Biomedical Science, University of Rochester Medical Center, Rochester, NY, 14642-0630, United States of America; d Department of Biostatistics and Computational Biology, University of Rochester, Rochester, NY, 14642-0630, United States of America; e Departments of Computational Medicine, Human Genetics, and Statistics, University of California, Los Angeles, CA, 90095-1554, United States of America

**Keywords:** Classification, Variable selection, Proximal algorithms, Distance majorization, *ℓ*_0_-constrained optimization

## Abstract

Modern biomedical datasets are often high-dimensional at multiple levels of biological organization. Practitioners must therefore grapple with data to estimate sparse or low-rank structures so as to adhere to the principle of parsimony. Further complicating matters is the presence of groups in data, each of which may have distinct associations with explanatory variables or be characterized by fundamentally different covariates. These themes in data analysis are explored in the context of classification. Vertex Discriminant Analysis (VDA) offers flexible linear and nonlinear models for classification that generalize the advantages of support vector machines to data with multiple classes. The proximal distance principle, which leverages projection and proximal operators in the design of practical algorithms, handily facilitates variable selection in VDA via nonconvex distance-to-set penalties directly controlling the number of active variables. Two flavors of sparse VDA are developed to address data in which instances may be homogeneous or heterogeneous with respect to predictors characterizing classes. Empirical studies illustrate how VDA is adapted to class-specific variable selection on simulated and real datasets, with an emphasis on applications to cancer classification via gene expression patterns.

## 1. Introduction

Advances in various technologies allow scientists to interrogate biological systems on a spectrum of organizational levels. Genomic and transcriptomic data are now particularly prevalent in the biomedical and life sciences. Regardless of the level of biological organization, modern data tend to be high-dimensional. It is generally recognized that analyzing small tractable subsets of the data is insufficient for proper inference. In addition to understanding how data are collected and generated, biomedical researchers traditionally had to possess enough domain knowledge to formulate well-motivated hypotheses and guess the important factors driving a measurement of interest. Owing to the complex evolutionary and biological processes underlying biomedical data, these guesses are now harder to make. Thus, variable selection looms large on the agenda of most statisticians confronted with such data, as modern data analyses typically amount to estimating sparse and/or low-rank structures from the data. Our focus is on sparsity in classification. Imposing low-rank structure is generally less scientifically informative than sparsity, which directly exposes the active predictors explaining a scientific phenomenon.

A few interesting examples of classification tasks in biomedical sciences include identifying gene expression patterns in microarrays of various human cancers (Dettling and Bühlmann, 2002; Guyon et al., 2002; Dettling, 2004; Weigelt et al., 2010), tumor prediction from imaging and histopathology data (Fuchs and Buhmann, 2011; Campanella et al., 2019), and disease diagnosis using electronic medical records (Xiao et al., 2018; Wang et al., 2019; Damotte et al., 2019). Our enumeration of applications and cited works is far from exhaustive. In each of the named applications, it is of interest to identify those predictors driving classification as well as to fit a robust classifier. Fortunately, biomedical scientists often have prior knowledge about *which* and *how many* predictors are linked to a response variable. For example, mutations in human genes such as the tumor suppressors *BRCA1* and *BRCA2* are associated with increased risk of breast cancer in women (Miki et al., 1994; Wooster et al., 1995). A tumor’s gene expression profile may also contain information about potential therapeutic targets, such as the *ERBB2* gene coding for receptor tyrosine-protein kinase erbB-2 (HER2 receptor) that is overexpressed in several classes of cancers, including subtypes of breast, ovarian, gastric, and lung cancers (Slamon et al., 1989; Konecny et al., 2003; Yan et al., 2015; Oh and Bang, 2020). Life science data are not uniquely high dimensional, but they serve as compelling motivation for investigating variable selection (Bühlmann et al., 2014).

Classification is undergirded by a rich literature and a variety of practical methods. Support Vector Machines (SVM) stand out in the universe of classification algorithms for their simplicity, interpretability, speed, and theoretical guarantees (Smola and Schölkopf, 1998; Vapnik, 1999). Although the value of SVM has been bolstered by fundamental advances in statistical theory and computer hardware, it is also challenged by the growing size of modern datasets. The Achilles heel of SVMs is their practical limitation to binary classification. Heuristics, such as one-versus-rest and one-versus-one, are common in software implementations (Fan et al., 2008; Chang and Lin, 2011). Given data containing multiple classes, these strategies rely on multiple binary SVMs to discriminate between classes. Regardless of the heuristic used, fitting multiple classifiers separately increases training time, obscures statistical interpretation, and violates the principle of parsimony. Moreover, it may be difficult to ascertain which heuristic is most appropriate for problems where one must balance training time against prediction quality. For example, one-versus-rest may result in a combined classifier with higher error rates than one-versus-one (Allwein et al., 2000) unless care is taken to carefully tune hyperparameters (Rifkin and Klautau, 2004). The interested reader may consult Lauer and Guermeur (2011) for a succinct summary of multiclass SVMs. A true multiclass SVM was developed by van den Burg and Groenen (2016). We will have more to say about this advance later.

In contrast, more traditional approaches in the literature successfully address multiclass data via a single model. Discriminant analysis is notable for its flexibility in both dimension reduction and classification. In particular, several theoretical and algorithmic extensions to sparse Linear Discriminant Analysis (LDA) were developed in the last decade; see for example Witten and Tibshirani (2011), Clemmensen et al. (2011), and Gaynanova et al. (2016). Sparse LDA in its many guises has the added benefit of associating predictors to classes. The class of $\ell_{1}$-regularized SVMs also has this capability under the one-versus-rest heuristic. In principle, addressing multiple classes directly and selecting predictors with strong associations to classes should improve prediction quality.

Vertex Discriminant Analysis (VDA) squarely addresses multiclass data (Lange and Wu, 2008), is compatible with a variety of penalties for variable selection (Wu and Lange, 2010), and can be extended to nonlinear classification (Wu and Wu, 2012). VDA is closely related to SVMs in the multiclass setting. Both GenSVM, the multiclass SVM of (van den Burg and Groenen, 2016), and VDA use vertex encodings based on $\left(c-1\right)$-simplices to represent c classes, apply decision rules based on proximity to vertices, and thus avoid ambiguous predictions compared to heuristic SVM methods (van den Burg and Groenen, 2016). The primary difference between VDA and GenSVM, as classifiers, is in how each relates a linear prediction $\hat{\text{y}}={\text{B}}^{⊤}\text{x}+{\text{b}}_{\text{0}}$ to the vertex set $Y=\left\{{\text{v}}_{\text{1}},{\text{v}}_{2},\ldots ,{\text{v}}_{c}\right\}$. VDA assigns each instance x with a vertex label y∈Y and computes distances between y and $\hat{\text{y}}$, whereas GenSVM projects $\hat{\text{y}}$ onto vectors ${\text{v}}_{j}-{\text{v}}_{k}$ normal to hyperplanes separating *each pair* of classes j and k. An $\ell_{1}$-penalized version of GenSVM might be able to select features via shrinkage, but it is unclear how one can extend the method to class-specific variable selection. We demonstrate how to adapt VDA so that it chooses a vertex encoding and leverages distance penalization (Chi et al., 2014) for this purpose.

Our main contributions are as follows:

1. We improve previous VDA algorithms by deriving a smooth, quadratic surrogate function. The Majorization-Minimization (MM) principle guarantees the descent property at each iteration and justifies the use of our local quadratic model. Minimization of the surrogate reduces to fast least squares.
2. For variable selection, we dispense with convex shrinkage-based penalties and instead impose explicit nonconvex $\ell_{0}$ constraints on the VDA loss. Distance majorization allows us to fold sparsity constraints into the loss as a convenient distance penalty. This action yields a greedy algorithm for variable selection. In particular, we illustrate the flexibility of our approach by showing how *structured sparsity* constraints, such as in class-specific variable selection, are readily incorporated in model fitting.
3. We provide empirical evidence of reliable support recovery in simulated and benchmark datasets. Our comparisons to previous VDA algorithms facilitate comparisons to several other classification and variable selection schemes. We also apply our method to a large real dataset on cancer gene expression.

The remainder of this text is organized as follows. Section 2 presents a smooth loss model for VDA that is readily approximated by a quadratic surrogate in the MM framework, reducing model fitting to solving a sequence of least squares problems. We then introduce distance penalization as a means to enforce constraints, particularly nonconvex $\ell_{0}$-type constraints. Section 3 comments on implementation, algorithmic convergence, cross validation, and other practical concerns. Section 4 describes and summarizes our numerical experiments on both simulated and real-world data. We offer a few concluding remarks on sparse VDA and other $\ell_{0}$-type estimators in Section 5.

## 2. Proposed methods

As a prelude to introducing our generic method, we first review VDA with 𝜖-insensitive pseudo-distances and derive a quadratic surrogate that both simplifies the associated empirical loss and converts minimization to iterative least squares. Next, we present a distance penalized version of VDA by introducing sparsity sets as a mechanism for variable selection. The section concludes with a brief remark on distance penalties applied to nonlinear VDA.

Here we clarify the notation used throughout this article. We restrict our attention to problems formulated over Euclidean space ${\mathbb{R}}^{p}$. Vectors are written in lowercase boldface, for instance y, and matrices are written in uppercase boldface, for instance X. The notation $\left\|\cdot \right\|$ indicates the Euclidean norm on the ambient space. In some cases we decorate a norm with a subscript, for example the to indicate the number Frobenius norm ${\left\|\cdot \right\|}_{F}$, to avoid ambiguity. Through abuse of notation, we may write ${\left\|\text{x}\right\|}_{0}=\sum_{j}1\left\{x_{j}\neq 0\right\}$ of nonzero components in a vector x. The superscript ⊤ indicates a vector or matrix transpose. The lowercase letter k denotes the number of selected parameters; equivalently, the lowercase letter s=1−k/p is reserved for the overall sparsity level. The maximum number of parameters p is understood from context. We reserve the bold symbols ${\text{e}}_{j}$ for denoting standard basis vectors in ${\mathbb{R}}^{p}$. In simulations we decorate objects representing the ground truth with a subscript asterisk, ${\text{B}}_{\text{*}}$. Finally, the letters m and t are reserved for inner iterations and outer iterations, respectively. For example, ${\text{B}}_{m}$ is an estimate at iteration m to the problem $\min_{\text{B}}f_{\rho \left(t\right)}\left(\text{B}\right)$ parameterized by a penalty coefficient $\rho \left(t\right)$, whereas ${\text{B}}_{t}$ denotes one of its solutions, either exact or approximate, at level t=0,1,2… and so on.

### 2.1. Majorizing ϵ-insensitive empirical risk

Given a dataset with n samples, p predictors, and c categories, the goal in VDA is to minimize the empirical risk

$$\frac{1}{n}\sum_{i=1}^{n}R\left({\text{y}}_{i},{\text{x}}_{i},\text{B}\right).$$

Here ${\text{x}}_{i}\in {\mathbb{R}}^{p}$ is the feature vector for sample i, ${\text{y}}_{i}\in {\mathbb{R}}^{c}$ is the sample’s label encoded as a vertex of a standard simplex in ${\mathbb{R}}^{c}$, and the entries of $\text{B}=\left(b_{jk}\right)$ are regression coefficients to be estimated. When c=2,3,or4, the simplex is a line segment, an equilateral triangle, or a regular tetrahedron, respectively. Note that our encoding of classes in vertex space has an extra dimension compared to the more parsimonious parameterization used in previous VDA models (Lange and Wu, 2008; Wu and Lange, 2010; Wu and Wu, 2012). For example, when c=3 the vertices of an equilateral triangle in ${\mathbb{R}}^{3}$ can be projected to ${\mathbb{R}}^{2}$ to reduce the number of free parameters. We deliberately choose the overparameterized version as it will be important later in developing class-specific variable selection. Specifically, we restrict vertex encodings to standard basis vectors ${\text{e}}_{j}\in {\mathbb{R}}^{c}$.

In any case, the predicted value for sample i’s class vertex ${\text{y}}_{i}$ is ${\text{B}}^{⊤}{\text{x}}_{i}$. We collect the rows ${\text{x}}_{i}^{⊤}$ into a feature matrix X and the rows ${\text{y}}_{i}^{⊤}$ into a class matrix Y. To fit XB to Y, we minimize the squared 𝜖-insensitive risk function

(1) $$\frac{1}{2n}\sum_{i=1}^{n}\max{\left\{0,{\left\|{\text{y}}_{i}-{\text{B}}^{⊤}{\text{x}}_{i}\right\|}_{2}-\epsilon \right\}}^{2}.$$

The residual ${\text{y}}_{i}-{\text{B}}^{⊤}{\text{x}}_{i}$ for sample i incurs no loss when ${\text{B}}^{⊤}{\text{x}}_{i}$ lies within a Euclidean ϵ-ball around its assigned vertex ${\text{y}}_{i}$. Otherwise, the residual contributes the reduced loss ${\left({\left\|{\text{y}}_{i}-{\text{B}}^{⊤}{\text{x}}_{i}\right\|}_{2}-\epsilon \right)}^{2}$ to the risk. If Y denotes a collection of c class vertices and $\hat{\text{B}}$ an estimated coefficient matrix, then an unassigned feature vector x is assigned to a class through the map

$$\text{x}\mapsto {\text{argmin}}_{\text{y}\in Y}\left\|{\text{y}}_{i}-{\hat{\text{B}}}^{⊤}{\text{x}}_{i}\right\|,$$

singling out the closest vertex. Inclusion of an intercept is beneficial when class numbers are unbalanced, and easily is achieved by adding an intercept row to B and a corresponding constant column 1 to X.

The squared ϵ-insensitive risk, ${\left\|\text{u}\right\|}_{\epsilon }^{2}=\max{\left\{0,\left\|\text{u}\right\|-\epsilon \right\}}^{2}$, is a differentiable alternative to its un-squared counterpart used in previous formulations of VDA (Wu and Lange, 2010). To leverage second-order information, we construct a quadratic surrogate in piecewise fashion based on the majorization (Lange, 2016)

$${\left\|\text{u}\right\|}_{\epsilon }^{2}\leq \left\{\begin{array}{ll} {\left\|\text{u}-\frac{\epsilon }{\left\|{\text{u}}_{m}\right\|}{\text{u}}_{m}\right\|}^{2}, & \left\|{\text{u}}_{m}\right\|>\epsilon \\ {\left\|\text{u}-{\text{u}}_{m}\right\|}^{2}, & \left\|{\text{u}}_{m}\right\|\leq \epsilon . \end{array}\right.$$

This complicated formula is an example of distance majorization (Chi et al., 2014). Indeed, ${\left\|{\text{u}}_{m}\right\|}_{\epsilon }=\text{dist}\left(\text{u},U_{\epsilon }\right)$ is best understood as the distance from u to $U_{\epsilon }$, the ball of radius ϵ centered at the origin. Easy calculations yield the projections $P_{U_{\epsilon }}\left({\text{u}}_{m}\right)=\frac{\epsilon }{\left\|{\text{u}}_{m}\right\|}{\text{u}}_{m}$ when ${\text{u}}_{m}\notin U_{\epsilon }$ and $P_{U_{\epsilon }}\left({\text{u}}_{m}\right)={\text{u}}_{m}$ when ${\text{u}}_{m}\in U_{\epsilon }$. In applying this majorization term-by-term to our VDA model (1), we set $\text{u}={\text{r}}_{i}={\text{y}}_{i}-{\text{B}}^{⊤}{\text{x}}_{i}$ and ${\text{u}}_{m}={\text{r}}_{mi}={\text{y}}_{i}-{\text{B}}_{m}^{⊤}{\text{x}}_{i}$ based on the residual of sample i. Thus, each term in the surrogate for our empirical risk model equals ${\left\|{\text{r}}_{i}-P_{U_{\epsilon }}\left({\text{r}}_{mi}\right)\right\|}^{2}$. This leads to the quadratic surrogate

(2) $$g\left(\text{B}|{\text{B}}_{m}\right)=\frac{1}{2n}\sum_{i=1}^{n}{\left\|{\text{z}}_{mi}-{\text{B}}^{⊤}{\text{x}}_{i}\right\|}^{2}=\frac{1}{2n}{\left\|{\text{Z}}_{m}-\text{XB}\right\|}_{F}^{2},$$

where XB predicts Y and Y is replaced by ${\text{Z}}_{m}$ with rows

$${\text{z}}_{mi}=\left\{\begin{array}{ll} {\text{x}}_{i}^{⊤}{\text{B}}_{m}, & \left\|{\text{y}}_{i}-{\text{B}}_{m}^{⊤}{\text{x}}_{i}\right\|\leq \epsilon \\ w_{mi}{\text{y}}_{i}^{⊤}+\left(1-w_{m,i}\right){\text{x}}_{i}^{⊤}{\text{B}}_{m}, & \left\|{\text{y}}_{i}-{\text{B}}_{m}^{⊤}{\text{x}}_{i}\right\|>\epsilon \end{array}\right.$$

$$w_{mi}=\frac{\left\|{\text{y}}_{i}-{\text{B}}_{m}^{⊤}{\text{x}}_{i}\right\|-\epsilon }{\left\|{\text{y}}_{i}-{\text{B}}_{m}^{⊤}{\text{x}}_{i}\right\|}.$$

In summary, our surrogate can be interpreted as an ordinary sum of squares with shifted responses.

### 2.2. Distance penalties, sparse projection, and variable selection

Having derived a quadratic surrogate for the empirical risk (1), we now develop our approach to variable selection. Following previous work on proximal distance algorithms (Chi et al., 2014; Xu et al., 2017; Keys et al., 2019), we penalize $f\left(\text{B}\right)$ by the squared Euclidean distance of B from a constraint set S. This creates the objective

(3) $$f_{\rho }\left(\text{B}\right)=f\left(\text{B}\right)+\frac{\rho }{2}\text{dist}{\left(\text{B},S\right)}^{2}$$

for ρ>0 large. If $P_{S}\left(\text{u}\right)$ denotes the Euclidean projection of u onto the closed set S, then we once again invoke the distance majorization $\text{dist}{\left(\text{u},S\right)}^{2}\leq {\left\|\text{u}-P_{S}\left({\text{u}}_{m}\right)\right\|}^{2}$, which can be combined with the surrogate (2) to yield

(4) $$g_{\rho }\left(\text{B}|{\text{B}}_{m}\right)=\frac{1}{2n}{\left\|{\text{Z}}_{m}-\text{XB}\right\|}_{F}^{2}+\frac{\rho }{2}{\left\|\text{B}-{\text{P}}_{m}\right\|}_{F}^{2}.$$

Here we understand ${\text{P}}_{m}$ as $P_{S}\left({\text{B}}_{m}\right)$ from context. Minimizing the penalized objective (3) via the surrogate (4) seeks a compromise between the empirical risk and the distance between estimated coefficients and their projection over the constraint set S. The strength of the proximal distance framework is now apparent as it allows one to incorporate various constraints provided a projection onto the constraint set S, $P_{S}\left(\cdot \right)$, is relatively inexpensive to evaluate. The sublevel sets $\left\{\text{x}:{\left\|\text{x}\right\|}_{2}\leq \lambda \right\}$, $\left\{\text{x}:{\left\|\text{x}\right\|}_{1}\leq \lambda \right\}$, and $\left\{\text{x}:{\left\|\text{x}\right\|}_{0}\leq k\right\}$ corresponding to Euclidean, lasso, and sparsity penalties are natural candidates for variable selection. Let us elaborate on the interesting case of sparsity constraints.

In classification with p features, one may attempt to select important variables by setting rows of the slope matrix B to zero so that feature j does not contribute to prediction; that is, $x_{ij}{\text{b}}_{j}={\text{0}}_{c}$. With this structure in mind, the structured sparsity set $S_{k}^{\text{row}}$ consists of those slope matrices with at most k nonzero rows

(5) $$S_{k}^{\text{row}}=\left\{\text{B}\in {\mathbb{R}}^{p\times c}:\sum_{j=1}^{p}1\left\{\left\|{\text{b}}_{j}\right\|\neq 0\right\}\leq k\;\text{where}\;\text{row}\;{\text{b}}_{j}\;\text{corresponds}\;\text{to}\;\text{feature}\;j\right\}.$$

Projection onto $S_{k}^{\text{row}}$ is achieved by ranking features according to their norms $\left\|{\text{b}}_{j}\right\|$ and then identifying the top k features via a partial sort, which requires $O\left(p+k\log k\right)$ comparisons once given the row norms. Omitted rows are then set to ${\text{0}}^{⊤}$. When an intercept ${\text{b}}_{0}$ is included in the model, the intercept is usually omitted in the constraint set. The intercept row is thus ignored in projection. In summary, projection onto a sparsity set amounts to computing order statistics on the row norms, $\left\|{\text{b}}_{j}\right\|$, and thresholding rows to select the highest ranking features.

Implicit in the preceding discussion is the assumption that all selected features are equally important in discriminating classes. Group penalties, such as the group lasso, come to mind in relaxing this assumption. We consider grouping coefficients into columns of the slope matrix B, each of which is associated to particular class. The sparsity set

(6) $$S_{k}^{\text{col}}=\left\{\text{B}\in {\mathbb{R}}^{p\times c}:{\left\|{\text{b}}_{\ell }\right\|}_{0}\leq k\;\text{for}\;\text{each}\;\text{column}\;{\text{b}}_{\ell }\right\},$$

restricts each column of a slope matrix B to have at most k nonzero components. In the context of VDA, the structure implied by $S_{k}^{\text{col}}$ is interpreted as selecting features associated with a class. One can further generalize by imposing a different model size restriction on each column. The logical extreme is the set

(7) $$S_{k}=\left\{\text{B}\in {\mathbb{R}}^{p\times c}:{\left\|\text{B}\right\|}_{0}\leq k\right\},$$

which simply selects k components of B without regards to row or column groupings of coefficients. Note that when $S=S_{k}^{\text{row}}$ or $S=S_{k}^{\text{col}}$ the hyperparameter k is naturally limited to values in $\left\{1,2\ldots ,p\right\}$ whereas the case $S=S_{k}$ has k take values in $\left\{1,2,\ldots ,cp\right\}$.

To conclude this section, we note that the special cases of sparsity constraint sets $S_{k}$, $S_{k}^{\text{row}}$, and $S_{k}^{\text{col}}$ reflect assumptions about the number of important features and class heterogeneity. The ideas presented here can easily be extended to Euclidean or lasso penalties on rows or columns via distance penalties. A notable benefit of the distance penalty approach is that it avoids differentiability issues; the penalty $\lambda^{-1}{\left\|\text{B}\right\|}_{1}$ is not differentiable whereas $\text{dist}{\left(\text{B},B_{\lambda }^{1}\right)}^{2}$, the distance to the centered $\ell_{1}$ ball of radius λ, is smooth. Our numerical experiments in later sections consider comparisons to lasso-type penalties.

### 2.3. Extension to nonlinear VDA

Linear VDA for feature selection easily generalizes to nonlinear VDA based on reproducing kernels. Construction of decision boundaries in sparse nonlinear VDA operates by selecting representative samples called support vectors in the SVM literature (Wu and Wu, 2012). Here we focus on defining and solving the optimization problem generated by nonlinear VDA. The goal is to estimate a classification vector $\text{ψ}\left(\text{x}\right)=\left[\psi_{1}\left(\text{x}\right),\psi_{2}\left(\text{x}\right),\ldots ,\psi_{c-1}\left(\text{x}\right)\right]$ by minimizing the objective ${\left(2n\right)}^{-1}\sum_{i=1}^{n}\max{\left\{\left\|{\text{y}}_{i}-\text{ψ}\left({\text{x}}_{i}\right)\right\|-\epsilon ,0\right\}}^{2}$. Given a reproducing kernel $W\left(\cdot ,\cdot \right)$, $\text{ψ}\left(\text{x}\right)$ assumes the functional form

$$\begin{array}{cc} \psi_{j}\left(\text{x}\right)=\sum_{i=1}^{n}b_{ij}W\left(\text{x},{\text{x}}_{i}\right)+b_{0j}, & j=1,2,\ldots ,\left(c-1\right) \end{array}$$

in predicting the vertex components from a feature vector x. Model parameters $\text{B}=\left(b_{ij}\right)$, including the intercepts $b_{0j}$, can be estimated by minimizing by criterion (3) with W substituted for X. Nonlinear prediction is achieved through the introduction of the reproducing kernel. The underlying model is still linear in its parameters. Explicitly, the loss and surrogate functions we use in the nonlinear case are

(8) $$f_{\rho }\left(\text{B}\right)=\frac{1}{2n}\sum_{i=1}^{n}\max{\left\{\left\|{\text{y}}_{i}-\text{ψ}\left({\text{x}}_{i},\text{B}\right)\right\|-\epsilon ,0\right\}}^{2}+\frac{\rho }{2}\text{dist}{\left(\text{B},S_{k}^{\text{row}}\right)}^{2}$$

(9) $$g_{\rho }\left(\text{B}|{\text{B}}_{m}\right)=\frac{1}{2n}{\left\|{\text{Z}}_{m}-\text{WB}\right\|}_{F}^{2}+\frac{\rho }{2}{\left\|{\text{P}}_{m}-\text{B}\right\|}_{F}^{2}.$$

Sparsity on the rows of B, induced by $S_{k}^{\text{row}}$, select at most k instances that contribute to a decision boundary. Alternatively, the heterogeneous group penalty $S_{k}^{\text{col}}$ allows VDA to select avatars specific to each group.

## 3. Algorithms

Let us briefly recall the MM principle (Lange, 2016) applied to the penalized objective (3). Given an anchor point ${\text{B}}_{m}$, the surrogate (4) majorizes (3) so that $f_{\rho }\left(\text{B}\right)\leq g_{\rho }\left(\text{B}|{\text{B}}_{m}\right)$ holds for every B in the essential domain of the penalized objective. At each iteration we select the unique stationary point ${\text{B}}_{m+1}$ minimizing the surrogate (4) around the anchor point ${\text{B}}_{m}$. These observations combined with the tangency condition reveal the chain of inequalities

$$f_{\rho }\left({\text{B}}_{m+1}\right)\overset{\text{majorization}}{\leq }g_{\rho }\left({\text{B}}_{m+1}\text{|}{\text{B}}_{m}\right)\overset{\text{definition}}{\leq }g_{\rho }\left({\text{B}}_{m}\text{|}{\text{B}}_{m}\right)\overset{\text{tangency}}{=}f_{\rho }\left({\text{B}}_{m}\right).$$

Thus, for fixed ρ, minimizing surrogate (4) guarantees the descent property and drives the penalized loss (3) downhill. Alternatively, it is enough to decrease $g_{\rho }\left(\text{B}|{\text{B}}_{m}\right)$ in cases where exact minimization is expensive and still preserve the logic of the inequalities above. The next two sections identify specific iteration schemes based on these principles. Additional details appear in Section A of the Supplementary Materials.

### 3.1. Direct minimization

The directional derivative of the surrogate (4) in the direction W is

$$d_{\text{W}}g_{\rho }\left(\text{B}|{\text{B}}_{m}\right)=-n^{-1}\text{tr}\left[{\left({\text{Z}}_{m}-\text{XB}\right)}^{⊤}\text{XW}\right]+\rho \text{tr}\left[{\left(\text{B}-{\text{P}}_{m}\right)}^{⊤}\text{W}\right].$$

At a stationary point $d_{\text{W}}g_{\rho }\left(\text{B}|{\text{B}}_{m}\right)$ vanishes for all W. It follows that

(10) $$\text{0}=-n^{-1}{\text{X}}^{⊤}\left({\text{Z}}_{m}-\text{XB}\right)+\rho \left(\text{B}-{\text{P}}_{m}\right)=\nabla g_{\rho }\left(\text{B}|{\text{B}}_{m}\right)$$

and that the B update is

(11) $${\text{B}}_{m+1}={\left[n^{-1}{\text{X}}^{⊤}\text{X}+\rho {\text{I}}_{p}\right]}^{-1}\left(n^{-1}{\text{X}}^{⊤}{\text{Z}}_{m}\text{+}\rho {\text{P}}_{m}\right).$$

To fit B across a spectrum of values for k and ρ, we extract the thin singular value decomposition (SVD) $\text{X}=\text{UΣ}{\text{V}}^{⊤}$ and invoke the Woodbury matrix identity

$${\left(\text{A}+\text{ECF}\right)}^{-1}={\text{A}}^{-1}-{\text{A}}^{-1}\text{E}{\left({\text{C}}^{-1}+\text{F}{\text{A}}^{-1}\text{E}\right)}^{-1}\text{F}{\text{A}}^{-1}\text{.}$$

This maneuver reduces the required matrix inversion to inverting a single diagonal matrix that depends on (a) the singular values Σ and (b) the values of n and ρ. This inverse only needs updating when ρ changes. Although the required thin SVD costs $O\left(nrp\right)$ operations, where $r\equiv \text{rank}\left(X\right)$, it is done only once. Thus, minimization of the penalized objective (3) via exact minimization of surrogate (4) is limited by the time and space complexities of one thin SVD and several matrix-matrix multiplication operations.

### 3.2. Steepest descent

One can take advantage of the quadratic form of (4) to derive a step size for gradient descent. The Taylor expansion

$$\begin{array}{c} g_{\rho }\left(\text{B}|{\text{B}}_{m}\right)=g_{\rho }\left({\text{B}}_{m}\text{|}{\text{B}}_{m}\right)+\text{tr}\left\{\nabla g_{\rho }{\left({\text{B}}_{m}\text{|}{\text{B}}_{m}\right)}^{⊤}\left(\text{B}-{\text{B}}_{m}\right)\right\}\\ +\frac{1}{2}\text{tr}\left\{{\left(\text{B}-{\text{B}}_{m}\right)}^{⊤}\nabla^{2}g_{\rho }\left({\text{B}}_{m}\text{|}{\text{B}}_{m}\right)\left(\text{B}-{\text{B}}_{m}\right)\right\}, \end{array}$$

is exact. In the particular case $\text{B}={\text{B}}_{m}-\gamma \nabla g_{\rho }\left({\text{B}}_{m}\text{|}{\text{B}}_{m}\right)$, elementary calculus leads to the steepest descent update

(12) $$\begin{array}{cc} \gamma_{m}=\frac{{\left\|{\text{G}}_{m}\right\|}_{F}^{2}}{n^{-1}{\left\|\text{x}{\text{G}}_{m}\right\|}_{F}^{2}+\rho {\left\|{\text{G}}_{m}\right\|}_{F}^{2}}, & {\text{B}}_{m+1}={\text{B}}_{m}-\gamma_{m}{\text{G}}_{m}, \end{array}$$

where ${\text{G}}_{m}=\nabla g_{\rho }\left({\text{B}}_{m}\text{|}{\text{B}}_{m}\right)$ is defined by formula (10).

### 3.3. Convergence criteria

Minimizing the penalized loss (3) with ρ>0 large is generally slow but is required to achieve optimality (Beltrami, 1970). We surmount the poor convergence characteristics of the penalty method by solving a sequence of subproblems (3) parameterized by an annealing schedule ${\left\{\rho \left(t\right)\right\}}_{t\geq 0}$. The idea is to choose an annealing schedule with $\rho \left(0\right)$ small (for example, $\rho \left(0\right)=1$) and gradually increasing $\rho \left(t\right)$ to a large maximum value (say 10^6^ to 10^8^). Unfortunately, the quality of the solution to the inner problem of minimizing the objective (3) for fixed $\rho \left(t\right)$ affects the quality of the outer solution where $\rho \left(t\right)$ reaches its maximum. It is therefore crucial to assess the quality of the inner solutions. These issues merit a brief discussion of convergence criteria.

Our previous work relied on the condition

$$\left|f_{\rho }\left({\text{B}}_{m+1}\right)-f_{\rho }\left({\text{B}}_{m}\right)\right|\leq \delta_{f}\left[1+f_{\rho }\left({\text{B}}_{m}\right)\right],$$

where $\delta_{f}$ is a control parameter that sets a minimum level of progress per inner iteration on a relative scale (Keys et al., 2019; Landeros et al., 2022). In retrospect, the gradient condition

$$\left\|\nabla f_{\rho }\left({\text{B}}_{m}\right)\right\|\leq \delta_{g},$$

is safer because convergence can be quite slow for large ρ even under Nesterov acceleration. The tangency condition of the MM principle conveniently allows one to compute the gradient $\nabla f_{\rho }\left({\text{B}}_{m}\right)$ through the equivalent expression $\nabla g_{\rho }\left({\text{B}}_{m}\text{|}{\text{B}}_{m}\right)$. Controlling the distance term across outer iterations t yields an approximate solution ${\text{B}}_{t}=\text{argmin}\;f_{\rho \left(t\right)}\left(\text{B}\right)$. Thus, we impose conditions on the distance penalty

$$\begin{array}{ccc} q_{t}\leq \delta_{d} & \text{or} & \left|q_{t}-q_{t-1}\right| \end{array}\leq \delta_{q},$$

where $q_{t}=\text{dist}\left({\text{B}}_{t},S_{k}^{\text{row}}\right)$ quantifies distance to the sparsity set. Here the control parameter $\delta_{d}$ defines a satisfactory level of closeness to $S_{k}^{\text{row}}$, and the dual criteria involving $\delta_{q}$ avoid excessive computing if progress slows dramatically. Our choices allow one to gradually guide solution estimates towards the constraint set while avoiding slow convergence due to large values of ρ. It is worth emphasizing that convergence is declared when both the gradient condition and the closeness condition are satisfied, regardless of whether $\rho \left(t\right)$ has hit its maximum.

### 3.4. Proximal distance iteration

Algorithm 1 summarizes the general minimization procedure in proximal distance iteration, including an early exit condition on the annealing path and Nesterov acceleration. Warm starts are implicit in Algorithm 1 as one moves from a $\rho \left(t\right)$-penalized subproblem to the next subproblem. The final projection step is justified when an approximate solution B is close to a sparsity set $S_{k}^{\text{row}}$ in Euclidean distance; that is, when feature selection finally stabilizes.

Let us now briefly address the theoretical convergence properties of Algorithm 1. The sparsity constraints described in Section 2.2 are defined by closed but nonconvex sets. In principle, projection operators onto nonconvex can be multivalued. For example, projection of the point (5, 2, 2, 3) onto the sparsity set $S_{3}$ is nonunique due to coordinate ties. Fortunately, the theoretical results from Keys et al. (2019) buttress Algorithm 1 under Zangwill’s global convergence theorem when the penalty coefficient ρ is fixed (Luenberger, 1984). First, $S_{k}^{\text{row}}$ is closed for any choice of k and nonempty. Second, the ϵ-insensitive loss $f\left(\text{B}\right)$ is coercive whenever the predictor matrix x has full rank. This implies the penalized loss $f_{\rho }\left(\text{B}\right)$ is also coercive. Finally, observe that the surrogate generated by distance majorization is related to the possibly multivalued proximal mapping (Parikh, 2014) by

$$\underset{{\text{P}}_{m}\in P_{S_{k}^{\text{row}}}\left({\text{B}}_{m}\right)}{\cup }{\text{argmin}}_{\text{B}}\left[f\left(\text{B}\right)+\frac{\rho }{2}{\left\|\text{B}-{\text{P}}_{m}\right\|}^{2}\right]={\text{prox}}_{\rho^{-1}f}\left[P_{S_{k}^{\text{row}}}\left({\text{B}}_{m}\right)\right].$$

Because set projections preserve closedness, it suffices to show that the projection operator $P_{S_{k}^{\text{row}}}\left({\text{B}}_{m}\right)$ is single-valued almost every-where to satisfy the hypothesis of Proposition 8 from Keys et al. (2019). For fixed ρ and k this guarantees that all limit points of the proximal distance iterates are stationary points, $\left\{\text{B}:\nabla f_{\rho }\left(\text{B}\right)=\text{0}\right\}$. In fact, convexity of the loss $f\left(\text{B}\right)$ guarantees that every surrogate $g_{\rho }\left(\text{B}|{\text{B}}_{m}\right)$ is ρ-strongly convex. The stronger conclusion of Proposition 11 from Keys et al. (2019) applies and allays concerns regarding selection of projected points when $P_{S_{k}^{\text{row}}}\left({\text{B}}_{m}\right)$ is set-valued.


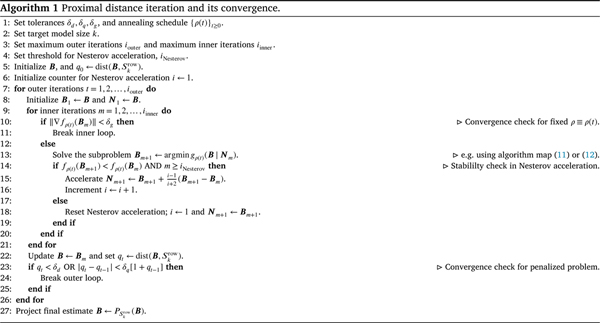


### 3.5. Tuning hyperparameters and probing stability

Before discussing implementation specifics, we note that the choice of sparsity set, either $S_{k}^{\text{row}}$ or $S_{k}^{\text{col}}$ as defined in (5) and (6), functions as a hyperparameter determining the set of selected variables. Changing the *size* of the target feature set k fundamentally changes the nature of the optimization problem. In contrast, perturbing the penalty constant in a lasso penalized model merely shifts or perturbs parameter estimates along a solution path (Hastie et al., 2001). Thus, model size is only indirectly determined by the penalty constants.

**Fig. 1.**
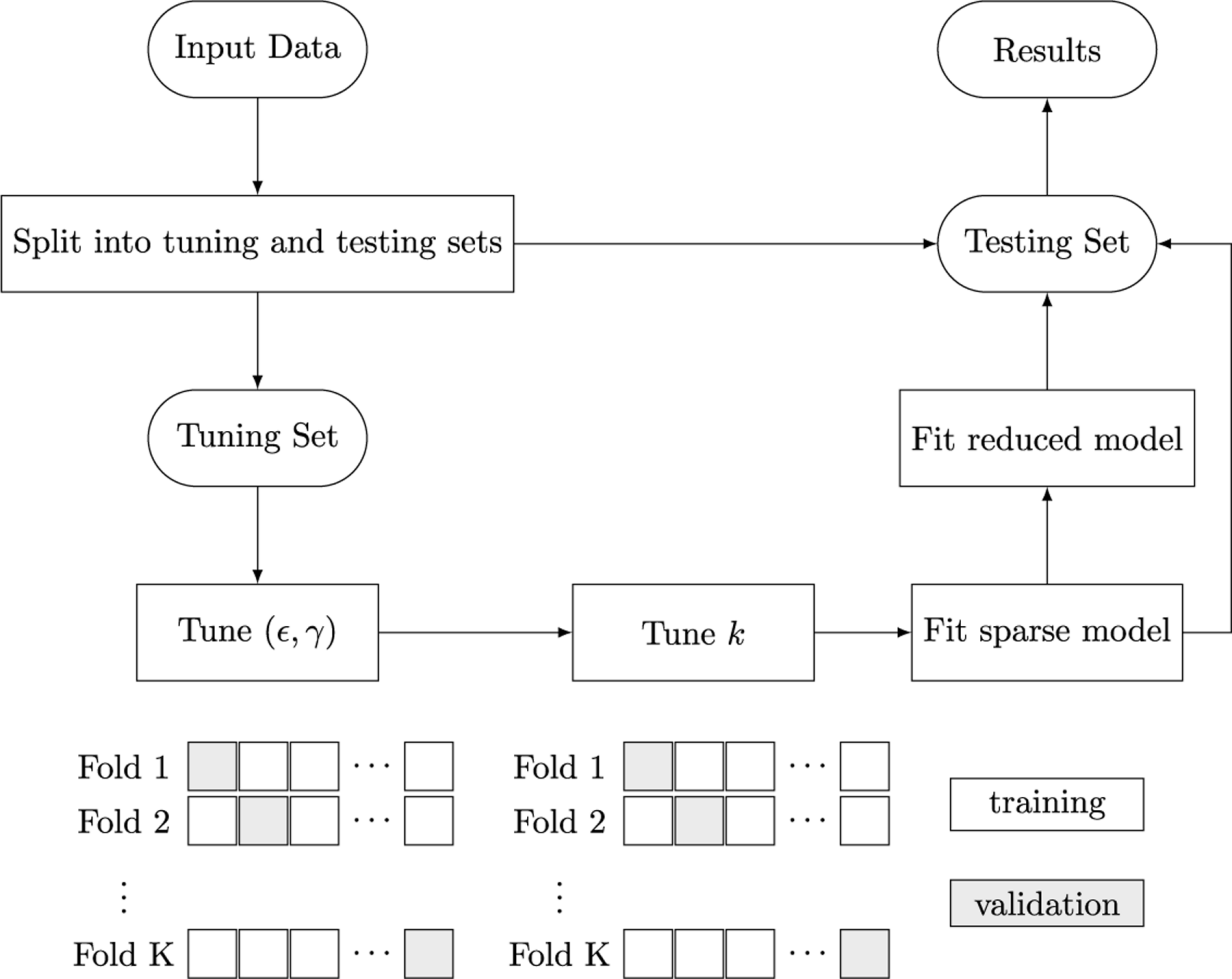
Cross validation for building a sparse VDA classifier.

Because a causal or optimal feature set is rarely known in advance, the effectiveness of our sparse VDA algorithms hinges on generating candidate feature sets and selecting optimal models. Cross validation is a practical way to evaluate the classification error of candidate models. We use a nested procedure as advocated by Cawley and Talbot (2010) which gives a realistic, albeit imperfect, assessment of model performance. For a given dataset ${\left\{\left({\text{y}}_{i},{\text{x}}_{i}\right)\right\}}_{i=1}^{n}$, we implement cross validation by partitioning the samples into two subsets, one for K-fold cross validation (the *tuning phase*) and one for evaluating out-of-sample prediction (the *test phase*). The former subset is further split into a *training set*, used to fit models under different hyperparameter settings, and a *validation set*, used to identify an optimal set of hyperparameters. Samples in the latter *testing set* never appear in the cross validation procedure. In the tuning phase, we propose a pair $\left(\epsilon ,\gamma \right)$, where γ is a kernel scale parameter in the nonlinear case, by sampling from a grid and fit a VDA classifier. Note that in the linear case we simply set γ=0.0. Each VDA classifier is then evaluated on a validation set so that a straightforward grid optimization can select the optimal pair based on the K-fold cross validation scores. Given an optimal pair $\left(\epsilon ,\gamma \right)$, we then sample the model size hyperparameter k from a grid and fit a sparse VDA classifier. Once again, we select an optimal model size k via grid optimization on classification accuracy. Ties in grid optimization should favor smaller values of k; that is, we select the more parsimonious model. Crucially, the same training and validation sets are used for tuning the two sets of hyperparameters. Fig. 1 summarizes our entire cross validation pipeline.

Repeated cross validation can be used to address stability in hyperparameters, the selected model size k, model parameter estimates, and the subset of selected features. Although the amount of computation involved seems onerous, our experience suggests that fast convergence of our VDA algorithms, even in the path following phase, is enough to make our proposed approach viable on a broad range of datasets.

## 4. Numerical experiments

In this section we demonstrate the feature selection capabilities of our VDA algorithms leveraging the proximal distance principle. Our first examples are simulation studies demonstrating how VDA reliably recovers sparse models without inflating classification errors. Next, we apply sparse VDA to classical microarray expression data (Dettling and Bühlmann, 2002) and compare the results to VDA subject to shrinkage-based penalties as described by Wu and Lange (2010). The remainder of this section benchmarks linear and nonlinear sparse VDA for datasets from the UCI Machine Learning Repository (Dua and Graff, 2019) and *Elements of Statistical Learning* (Hastie et al., 2001). We highlight how class-specific variable selection improves classification accuracy and produces tighter interval estimates of the true number of relevant features, particularly in fitting sparse VDA to RNA expression profiles of tumors in The Cancer Genome Atlas (TCGA) data. Table 1 summarizes the real datasets used for our benchmarks. Examples drawn from the UCI Machine Learning Repository are not high-dimensional but well-known, well-understood, and thus serve as sanity checks. High-dimensional examples are drawn exclusively from cancer datasets. Additional details on the real and simulated data are available in Section B of the Supplementary Materials.

Our empirical studies compare sparsity-based and shrinkage-based approaches to variable selection. Specifically, we test VDA with the homogeneous, $S_{k}^{\text{row}}$, and heterogeneous, $S_{k}^{\text{col}}$, group variable selection constraints (5) and (6), respectively and compare with their convex analogues

(13) $$C_{\lambda }=\left\{\text{B}\in {\mathbb{R}}^{p\times c}:\sum_{i,j}\left|{\text{B}}_{ij}\right|\leq \lambda \right\},$$

(14) $$C_{\lambda }^{\text{row}}=\left\{\text{B}\in {\mathbb{R}}^{p\times c}:{\left\|{\text{b}}_{j}\right\|}_{1}\leq \lambda \;\text{where}\;\text{row}\;{\text{b}}_{j}\;\text{corresponds}\;\text{to}\;\text{feature}\;j\right\},$$

and

(15) $$C_{\lambda }^{\text{col}}=\left\{\text{B}\in {\mathbb{R}}^{p\times c}:{\left\|{\text{b}}_{\ell }\right\|}_{1}\leq \lambda \;\text{for}\;\text{each}\;\text{column}\;{\text{b}}_{\ell }\right\}.$$

Although $C_{\lambda }^{\text{row}}$ differs significantly from $S_{k}^{\text{row}}$, both constraint sets assume a given feature plays a similar role across groups. We refer to VDA with constraints (5) and (6) as VDA_HomL0_ and VDA_HetL0_, respectively. Here the abbreviations “Hom” and “Het” emphasize whether the model assumes homogeneous or heterogeneous classes. Similarly, VDA with constraints (13), (14), and (15) are dubbed VDA_L1_, VDA_HomL1_, and VDA_HetL1_, respectively. Lastly, we note that nonlinear VDA uses a Gaussian kernel (Radial Basis Function Kernel) in all our examples.

### 4.1. Simulation studies of sparse recovery

Let us first investigate the numerical performance of sparse VDA methods, VDA_HomL0_ and VDA_HetL0_, using simulated data. In both cases, we consider p=1000 predictors with multivariate normal distribution, $\text{x}∼N\left(\text{0},\text{Σ}\right)$, such that the covariance matrix Σ follows a Toeplitz structure, $\Sigma_{ij}=\rho^{\left|i-j\right|}$. Of the 1000 predictors, only a small subset J of size $k_{\text{*}}\equiv \left|J\right|=30$ factors into establishing the ground truth for class labels. Specifically, we randomly sample a slope matrix ${\text{B}}_{\text{*}}\in {\left\{-1,0,1\right\}}^{p\times c}$ with exactly $k_{\text{*}}=30$ nonzero rows, each corresponding to an informative variable, and assign classes via the decision rule

$${\text{x}}_{i}\mapsto {\text{argmin}}_{\text{y}\in G}\left\|{\text{y}}_{i}-{\text{B}}_{*}^{⊤}{\text{x}}_{i}\right\|,$$

where we remind the reader that Y is the standard simplex in ${\mathbb{R}}^{c}$ with standard basis vectors, ${\text{e}}_{i}$, as vertices. The vertex label for each instance ${\text{x}}_{i}$ is denoted ${\text{y}}_{i}$ and is arranged as the i-th row in a vertex label matrix $\text{Y}\in {\left\{0,1\right\}}^{n\times c}$. Unfortunately, ${\text{B}}_{\text{*}}$ is not necessarily the best fitting slope matrix since each prediction ${\text{B}}_{*}^{⊤}{\text{x}}_{i}$ has expectation $\text{0}\in {\mathbb{R}}^{c}$. We remedy this defect by computing a sparse shift matrix $\text{S}\in {\mathbb{R}}^{n\times p}$ which satisfies the linear system

$${\text{S}}_{\text{J}}{\text{B}}_{\text{*},\text{J}}=\left(\text{Y}-\text{X}{\text{B}}_{\text{*}}-\text{E}\right),$$

over the support set J and update X as X↦X+S. This modification shifts the expectation of ${\text{B}}_{*}^{⊤}{\text{x}}_{i}$ towards the associated label ${\text{y}}_{i}$. The noise matrix $\text{E}\in {\mathbb{R}}^{n\times c}$ with independent and identically distributed entries ${\text{E}}_{ij}∼N\left(\text{0},\sigma^{2}\right)$ allows us to control the extent to which ${\text{B}}_{\text{*}}$ and its support J reflect the ground truth. Our simulations compute $\sigma^{2}$ via its relationship to the Signal-to-Noise Ratio (SNR)

$$\sigma^{2}=\frac{\frac{1}{c}\sum_{j=1}^{c}\text{Var}\left({\text{y}}_{\text{*},j}\right)}{\text{SNR}},$$

in which ${\text{y}}_{\text{*},j}$ is column 𝑗 of the vertex label matrix $\text{Y}\in {\left\{0,1\right\}}^{n\times c}$.

Our simulation studies vary the number of samples n∈{500,2000}, the number of classes $c\;\in \left\{3,10\right\}$, the Toeplitz matrix parameter $\rho \;\in \left\{0,1,0.5,0.9\right\}$, and the $\text{SNR}\;\in \left\{0.1,1.0,10.0\right\}$. These choices help us investigate the performance of VDA-based classifiers across a diverse set of scenarios, which consider underdetermined problems, correlations between predictive and noise variables, and over-lapping classes. To illustrate the predictive and variable selection capabilities of VDA with sparsity set constraints, our benchmarks report the following metrics

- the true positive rate based on the ground truth ${\text{B}}_{\text{*}}$ established by our simulation model,
- the ratio of true positives to predicted positives (positive predictive value),
- out-of-sample classification errors based on a test set with 1000 samples, and
- timing results that reflect total time spent to identify a support and fit a final model.

Under the assumption that a sparse support exists, our aim is to show that sparse linear VDA models reliably identify a small set of informative predictors while accurately predicting classes. By considering underdetermined and overdetermined regimes under different SNR and correlation levels, our results suggest a degree of consistency in recovering sparse supports in interesting and challenging scenarios pertinent to biomedical science applications.

#### 4.1.1. Homogeneous classes

We first consider structured sparsity in a setting in which distinct classes are predicted by the same set of features. In this setting, we say that classes are *homogeneous* in the sense that they share a common support set J such that the linear prediction $\text{X}{\text{B}}_{\text{*}}$ reflects the ground truth under the VDA model. We compare VDA with the sparse, nonconvex HomL0 penalty and shrinkage-based, convex L1 penalty against Multi-Group Discriminant Analysis (MGSDA) (Gaynanova et al., 2016) and a L1-regularized SVM (L1R-SVM) as implemented in LIBLINEAR (Fan et al., 2008). Notably, sparsity in MGSDA relies on shrinkage due to a Euclidean norm penalty rather than a lasso. For the VDA models, we consider a predictor j to be selected if its corresponding row ${\text{b}}_{j}$ in the fitted slope matrix $\hat{\text{B}}$ has nonzero norm. MGSDA and L1R-SVM have analogous selection criteria, as established in relevant literature and documentation. The reader should note that our VDA methods are implemented in the Julia programming language, whereas as MGSDA is provided as an R package and LIBLINEAR is a C library accessed via a Julia wrapper.

Table 2 reports timing results and classification error rates for simulated classification problems under the homogeneous class assumption. The VDA timing results suggest an approximately linear relationship with the number of samples. This is expected behavior since matrix multiplication by $\text{X}\in {\mathbb{R}}^{n\times p}$ is among the most frequently occurring operations in our algorithms. The convex version of sparse VDA, VDA_L1_, is often the fastest method overall. Results for MGSDA mostly align with the VDA models except in the cases with n=2000 and c=10 which we attribute to a larger number of iterations taken to fit the multiclass data. L1R-SVM appears as the slowest method due to it fitting multiple binary SVMs; this is an embarrassingly parallel problem and thus is easily improved. Readers should not draw strong inferences from these relative rankings as the results may easily change were one to implement every method in a common programming language and a common software design. Yet, the speed advantage of a single classifier (VDA and MGSDA) over a multi-classifier approach (SVM) is reflected in Table 2 as is well-established in literature. The apparent linear scaling of VDA with *n* will break down in the n≫p regime as the required singular value decomposition of X will come to dominate the cost of our MM algorithms, specifically as the storage costs for X and its decomposition overwhelm memory resources on a computer. A similar claim holds for 𝑝 and the p≫n regime. Hence, the current implementation of sparse VDA is limited by the cost of extracting a thin SVD.

Error rates in Table 2 are crucial in establishing validity of our simulation study. In the absence of theory, we require at a minimum that error rates decrease towards 0 as the number of samples *n* and the SNR increase. For example, the median classification error for MGSDA decreases from 57% to 0% as SNR increases from 0.1 to 10 in the setting with n=2000, c=3, and ρ=0.1. The trend also holds moving from n=500 to n=2000 across each scenario. Increasing SNR in the underdetermined regime $\left(n=500\right)$ has apparently less influence on classification accuracy. This is expected because the underdetermined regime represents a challenging scenario in which classifiers are prone to overfitting. Surprisingly, the advantage of sparse VDA reemerges if one considers SNR and ρ simultaneously. With n=500 and c=3 fixed, VDA_HomL0_ improves median error rates from 66% to 0% as both SNR and ρ increase whereas error rates remain mostly unchanged for VDA_L1_, MGSDA, and L1R-SVM. In fact, increasing ρ alone appears to improve classification error rates. This result can be understood by considering that, while noise variables become strongly correlated with informative predictors, some informative predictors also become strongly correlated with one another.

**Fig. 2.**
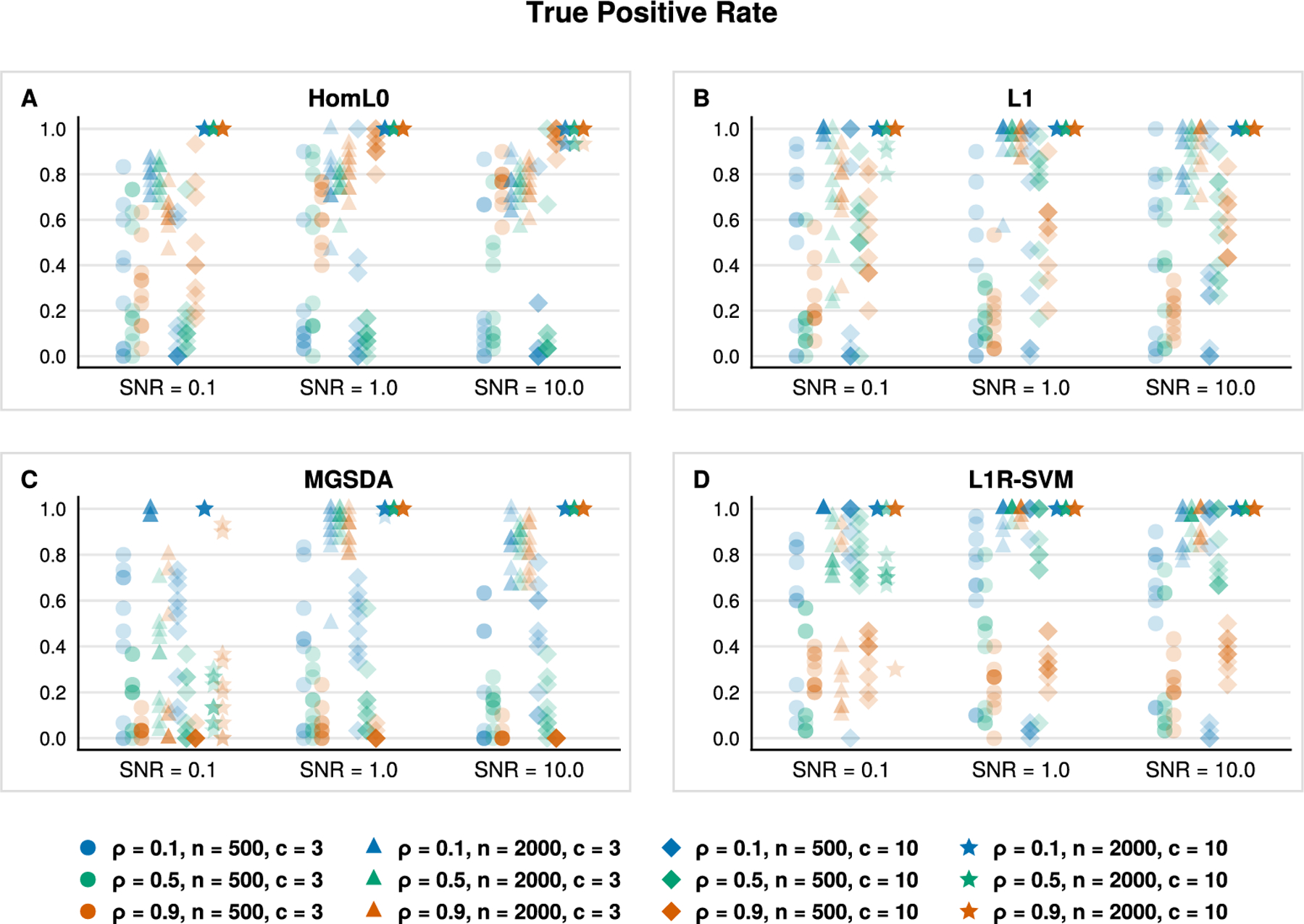
True positive rates (*y*-axis) for variables selected by VDA_HomL0_, VDA_L1_, MGSDA, and L1R-SVM, based on 10 simulation replicates in each scenario. Here $\rho \in \left\{0.1,0.5,0.9\right\}$ is the parameter in the Toeplitz covariance matrix $\sum_{ij}=\rho^{\left|i-j\right|}$ used to simulate each instance as $\text{x}∼N\left(\text{0},\sum \right)$. Classes are homogeneous in the sense that a common subset of variables of size $k_{*}=30$ determine class assignments. (For interpretation of the colors in the figure(s), the reader is referred to the web version of this article.)

**Fig. 3.**
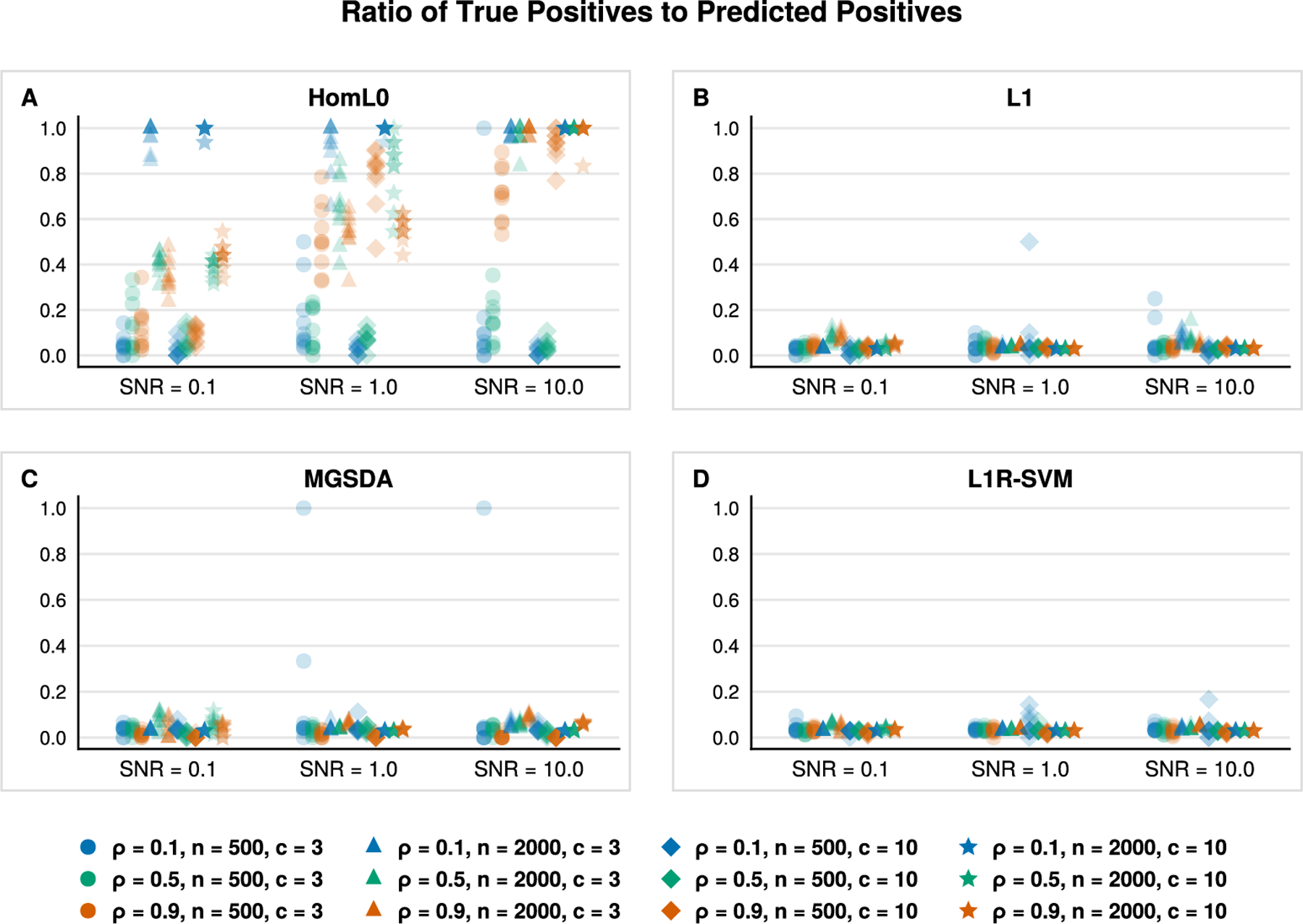
Ratio of true positives and predicted positives for variables (*y*-axis) selected by VDA_HomL0_, VDA_L1_, MGSDA, and L1R-SVM, based on 10 simulation replicates in each scenario. Classes are homogeneous in the sense that a common subset of variables of size $k_{*}=30$ determine class assignments.

Fig. 2 and Fig. 3 aid us in validating the previous claim. Fig. 2 reports the true positive rate with respect to the ground truth sparse support. The true positive rate for all classifiers improves as both *n* and SNR increase as one might anticipate. This sanity check is reflected in Fig. 2 by noting the progressions along the *x*-axis (left to right), from circles to triangles, and from diamonds to stars in each subplot. The Euclidean and lasso penalties have an edge over our HomL0 method in terms of identifying true positives (see blue circles and triangles). However, Fig. 3 clarifies the advantage of the $\ell_{0}$ approach. Examining the positive predictive value of each classifier aids us in distinguishing between a method that may incorporate noise variables to improve prediction over those that recover the ground truth support. VDA with sparsity constraints, VDA_HomL0_, is appreciably more sensitive and specific than competing methods in identifying the ground truth sparse support. For example, the underdetermined regime $\left(n=500\right)$ is challenging for VDA_HomL0_ when the SNR equals 0.1 (Figure 3A, circles and diamonds). Yet, our method is aided by increasing the correlation strength between all predictors from ρ=0.1 to ρ=0.9. In the overdetermined regime $\left(n=2000\right)$, a high degree of correlation obscures support recovery unless the underlying signal is strong (Figure 3A, 3B, orange markers). Comparing VDA_HomL0_ against VDA_L1_ further clarifies these results as the two methods differ only in the choice of penalty. The L1 version of VDA shrinks the fitted slope matrix $\hat{\text{B}}$ so that the linear predictions based solely on the ground truth support J, ${\text{X}}_{J}{\hat{\text{B}}}_{J}$, are closer to the origin rather than the underlying simplex vertices. Thus, spurious predictors enter into the model’s predictions to compensate. In contrast, VDA_HomL0_ avoids shrinkage and does not incorporate spurious predictors through the same mechanism. Cross-referencing Fig. 3 against Table 2 suggests that our sparse VDA classifiers are both parsimonious and predictive. The difference in performance is stark in comparing error rates in the underdetermined regime with correlated predictors, a setting that is relevant to genomic applications. Notably, the shrinkage-based methods maintain an advantage in prediction accuracy when the signal-to-noise ratio is low (SNR = 0.1).

#### 4.1.2. Heterogeneous classes

**Fig. 4.**
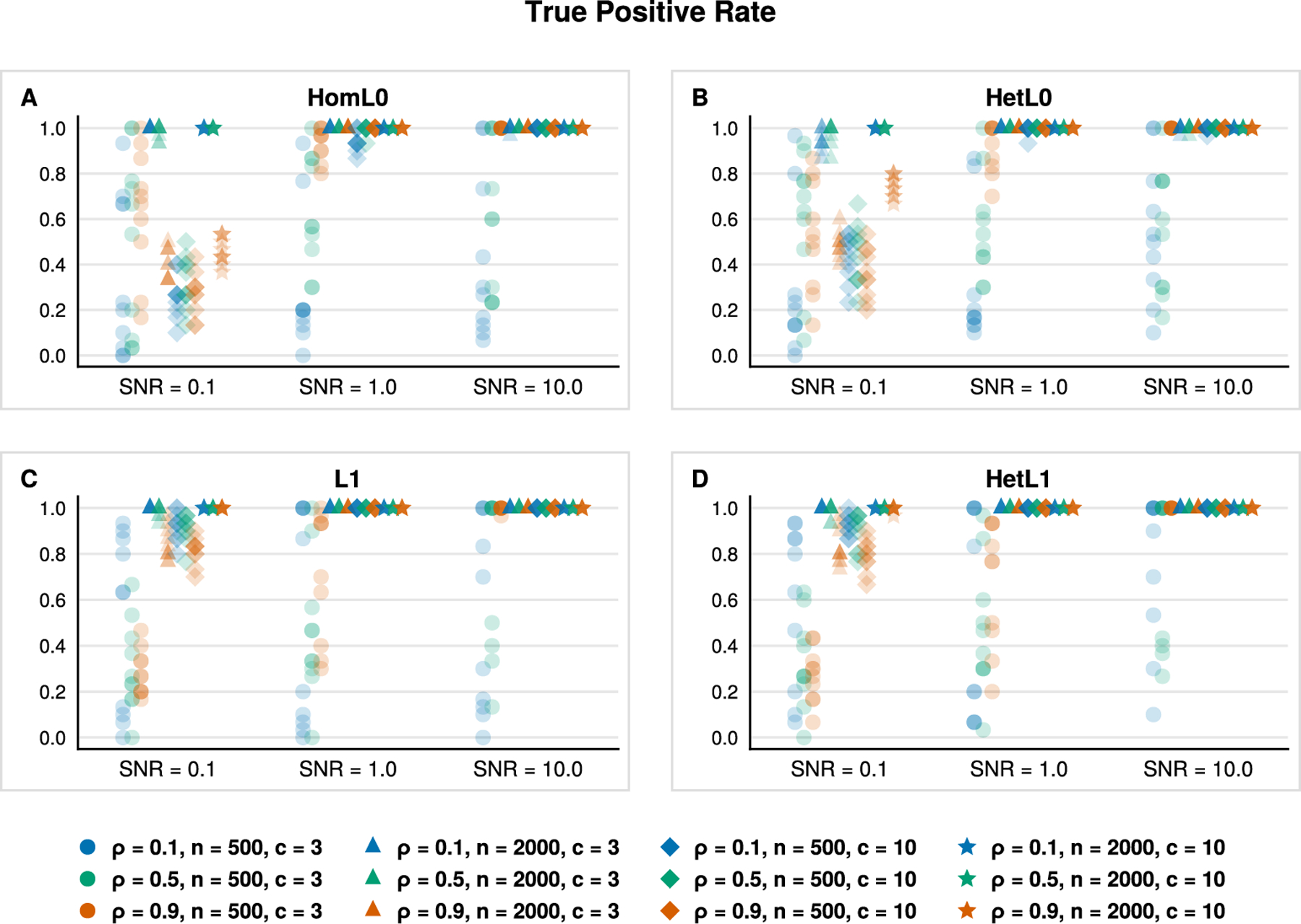
True positive rates per class (*y*-axis) for variables selected by VDA_HomL0_, VDA_HetL0_, VDA_L1_, and VDA_HetL1_, based on 10 simulation replicates in each scenario. Classes are heterogeneous in the sense that each class is determined by a subset of variables of size $k_{*}=\left\lfloor 30/c\right\rfloor$. True positives are assessed within each class and then averaged over classes to arrive at the reported values.

**Fig. 5.**
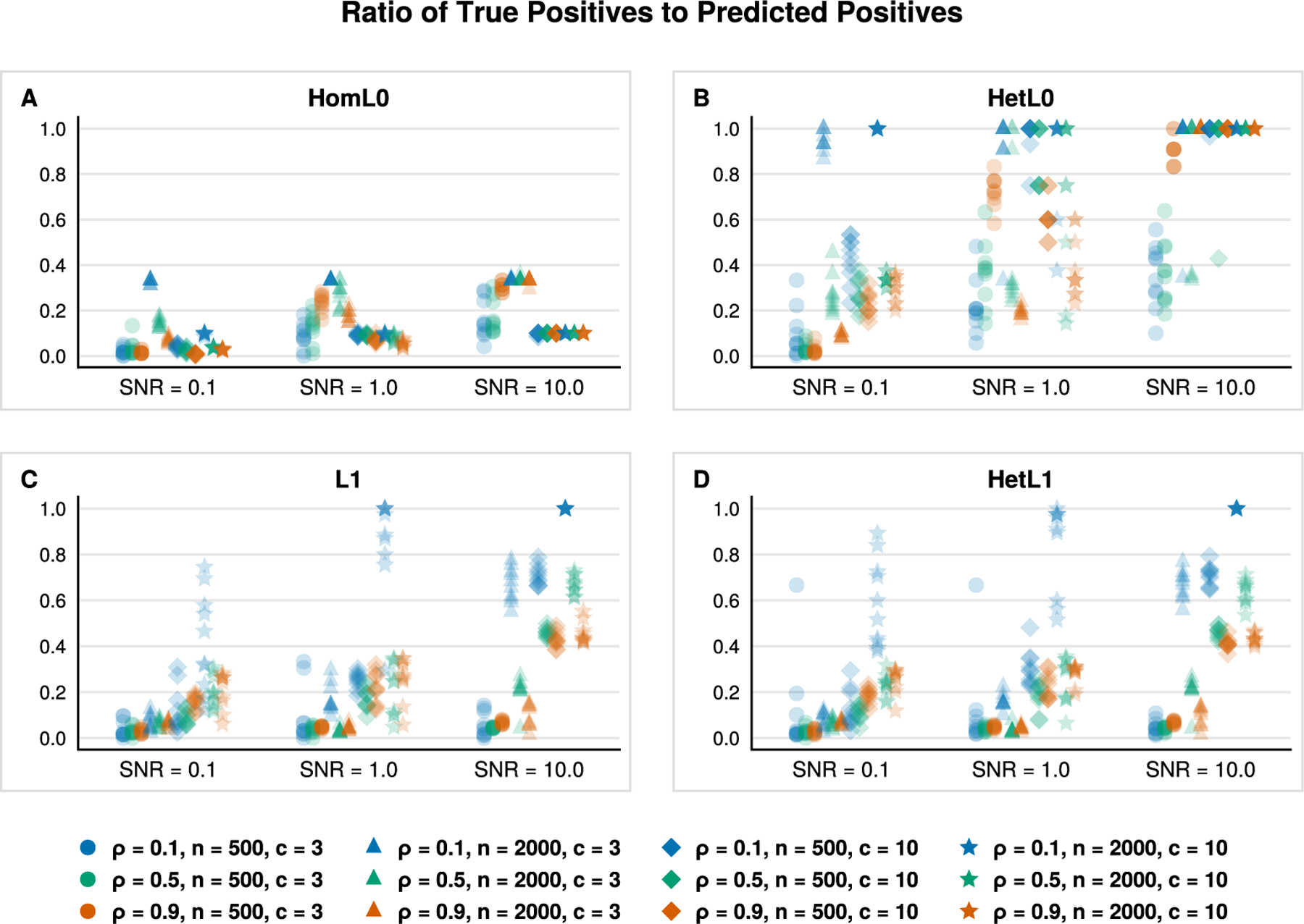
Per class ratio of true positives and predicted positives for variables (*y*-axis) selected by VDA_HomL0_, VDA_HetL0_, VDA_L1_, and VDA_HetL1_ based on 10 simulation replicates in each scenario. Classes are heterogeneous in the sense that each class is determined by a subset of variables of size $k_{*}=\left\lfloor 30/c\right\rfloor$. True and predicted positives are assessed within each class and then averaged over classes to arrive at the reported values.

Next, we exploit the flexibility of distance penalties to investigate class-specific variable selection. Our simulations now consider *heterogeneous* classes in the sense that each class has a possibly distinct set of predictive variables. Given a size $k_{*}$ of true, informative variables we randomly assign $\left\lfloor k_{*}/c\right\rfloor$ variables to each class so that ${\text{B}}_{*}^{⊤}\text{x}$ is close to vertex ${\text{e}}_{\ell }$ whenever instance x belongs to class ℓ. The simulation model is identical to that of the homogeneous setting except for the assignment of informative variables to each class. We compare four VDA models with distance penalties based on HomL0 (Equation (5)), HetL0 (Equation (6)), L1 (Equation (13)), and HetL1 (Equation (15)). Table 3 reports timing results and classification error rates which mirror that of Table 2. The shrinkage-based penalties, L1 and HetL1, are superior to our sparsity-based penatlies in the low SNR setting. However, the shrinkage-based VDA methods generally select additional variables outside the ground truth support as reflected in Fig. 4 and Fig. 5. In particular, Fig. 5 illustrates that aggregate positive predictive value of our proposed class-specific method, VDA_HetL0_, generally outperforms the structured and unstructured shrinkage methods VDA_HetL1_ and VDA_L1_, respectively. The HomL0 penalty explicitly assumes a homogeneous class structure and is thus prone to selecting extraneous predictors within each class.

### 4.2. Comparison with previous VDA methods

**Fig. 6.**
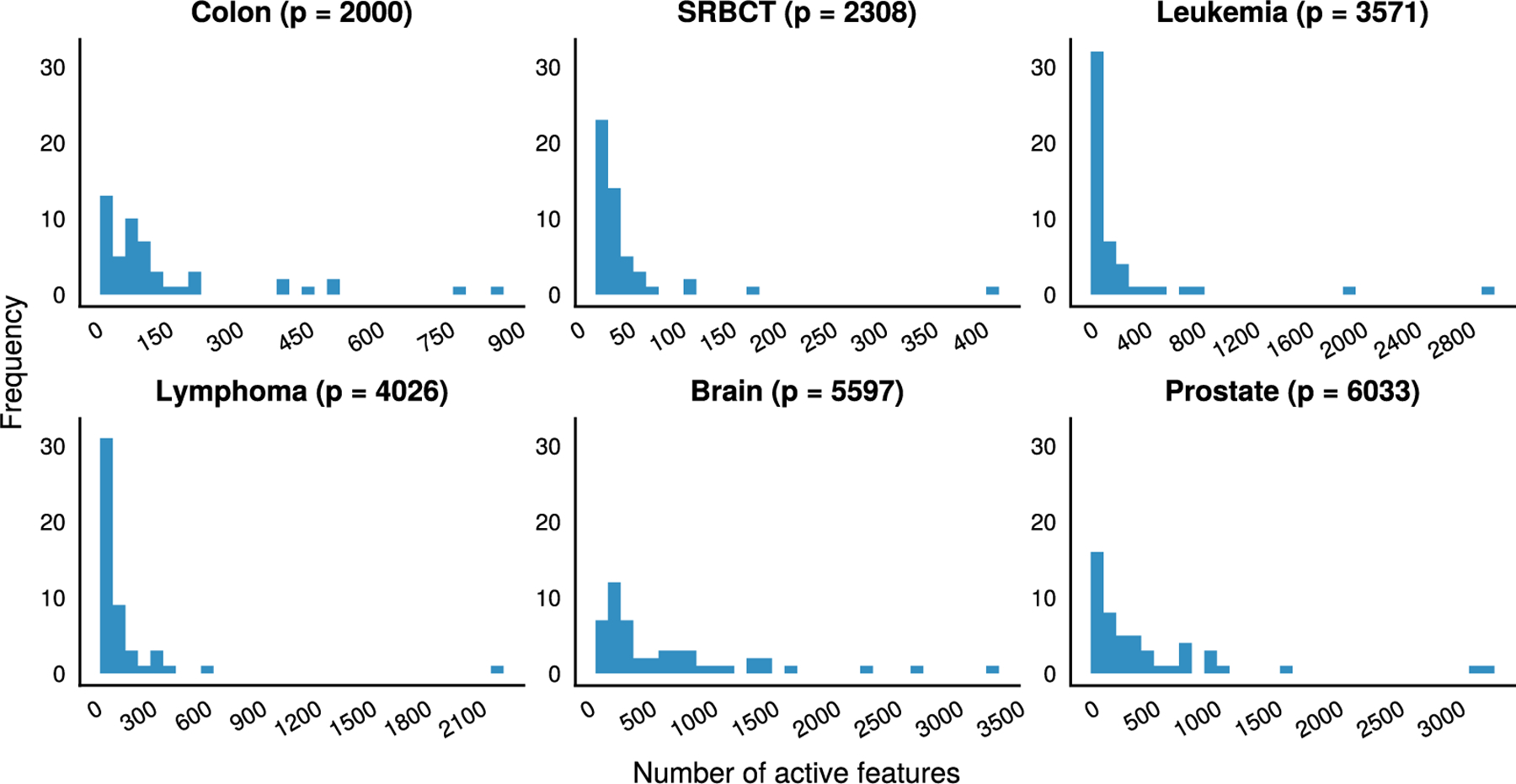
Distribution of optimal model sizes identified by sparse VDA using the HetL0 constraint (6), based on 50 replicates.

Our next set of experiments consider applications to cancer microarray data, where it is common for the number of features to dominate the number of samples, p≫n. Here Table 4 reports results for distance-penalized linear VDA classifiers across 6 datasets for different cancers previously analyzed by Dettling and Bühlmann (2002). The **leukemia**, **prostate**, and **colon** datasets involve two classes while the rest involve multiple classes. Our error rates are similar to previous results for VDA_LE_ (Wu and Lange, 2010), which uses a hybrid penalty combining lasso and Euclidean penalties, and hierarchical clustering (Dettling and Bühlmann, 2002). Although our cross validation errors are not directly comparable to the best leave-one-out errors noted in Table 7 of Dettling and Bühlmann (2002), we succeed in selecting models with fewer active genes than reported in Table 9. The left-skewed distributions in Fig. 6 support this contention in spite of the wide ranges reported for **brain** and **prostate**. While the selected subset size distribution for the sparsity-based methods is wider compared to VDA_LE_, we see that class-specific variable selection yields tighter intervals and improves classification accuracy. VDA_LE_ outperforms our VDA models on the **colon** and **prostate** examples in part due to its loss function, which is robust to outliers by virtue of not squaring errors, and its penalty, which interpolates between lasso and Euclidean penalties.

### 4.3. Linear classifier benchmarks

We now turn our attention to fitting linear classifiers to various datasets from the UCI Machine Learning Repository (Dua and Graff, 2019). In each example, we take care to fit VDA classifiers across a spectrum of possible model sizes k that cover about 50% of the space on a logarithmic scale. We note that, except for the **TCGA-HiSeq** and **HAR** datasets, generating 50 cross validation replicates requires a few minutes of computing. Table 5 records our results, which includes comparisons to L1-regularized SVM using the One-Versus-Rest (OVR) approach to multiclass data. As a reminder, an SVM using OVR requires *c* SVMs to classify data on *c* classes. It is known that SVM-OVR is sensitive to hyperparameter tuning (Rifkin and Klautau, 2004) so we consider our classification results comparable to VDA. A merit of our sparse VDA algorithms is the fact that it deals with all class simultaneously in a single model and is thus easy to fit without parallel processing beyond BLAS and more interpretable.

A few examples are noteworthy. Interestingly, most of the smaller datasets such as **iris** can be fitted with fewer features. In contrast, the **letters** example consistently fits with all 16 features, which were selected by experts to aid in letter recognition. The features in the **splice** dataset are 60 sites on a DNA molecule occupied by a T, C, G, or A nucleotide, represented via one-hot coding per site. This gives a total of 240 features. Our sparsity-based VDA classifiers successfully identify sequences as intron-exon, exon-intron, or nonsplice junctions using only 10 to 35 sites. In the **optdigits** example, it is conceivable that features from the dataset, which summarize white pixel counts of 32 × 32 images along 4×4 blocks, are in fact redundant. This explains why our results indicate that roughly 55 to 62 out of 64 pixel values are needed to correctly discriminate most of the digits in the 1797 test samples. In the higher dimensional examples **HAR** and **TCGA-HiSeq**, we note that the class-specific, shrinkage-based method HetL1 struggles to eliminate features compared to our sparsity-based methods, HomL0 and HetL0. This is most pronounced in the RNA expression data where one expects only a small subset of genes to be relevant in each cancer and thus suggests that the sparsity-based VDA classifiers have better control over false positives compared to shrinkage-based methods. Note that we report more selected variables for HetL0 (516 genes) and HetL1 (6449 genes) compared to HomL0 (326 genes) because the former two select distinct, potentially non-overlapping sets for each class. Dividing the values for HetL0 and HetL1 by the number of classes c=5 suggests the shrinkage-based penalty is in fact selecting more variables across classes.

**Fig. 7.**
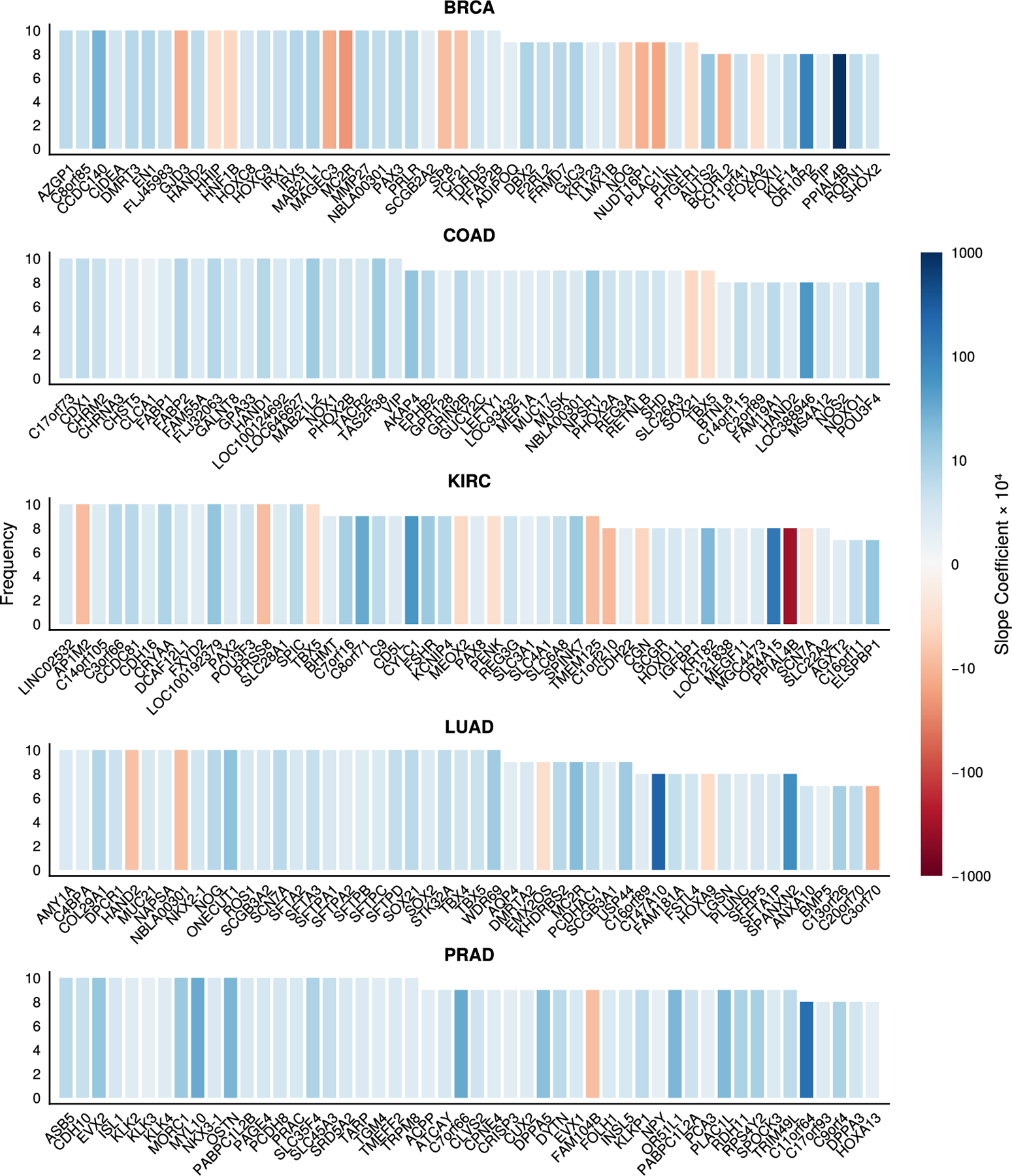
Occurrence of the top 50 selected genes within each cancer in **TCGA-HiSeq**, based on 10 cross validation replicates. Gene symbols appear along the 𝑥-axis and their corresponding frequency in cross validation is represented by the *y*-axis. The magnitude and sign of each gene’s slope in VDA is represented in color, with red indicating negative slopes and blue indicating positive slopes.

Lastly, we are pleased that sparse VDA performs well on the **TCGA-HiSeq** dataset. This dataset contains gene expression patterns in 801 tumors measured by RNA sequencing technology. Tumors are labeled as one of five types of cancers: breast invasive carcinoma (BRCA), kidney renal clear cell carcinoma (KIRC), colorectal adenocarcinoma (COAD), lung adenocarcinoma (LUAD), and prostate adenocarcinoma (PRAD). In this setting, it is expected that only a subset of genes serves to discriminate between cancer types. Our VDA algorithms reliably select anywhere between 57 to 2336 genes out of the entire set of 20264. To better understand how class-specific variable selection aids VDA in classification, we examine the selected gene subsets across 10 cross validation replicates. For a given subset within a class, we rank each gene by how frequently it is replicated in cross validation. As Fig. 7 illustrates, roughly 25 genes are selected consistently within each cancer class. It is likely that the less replicated genes are subject to some degree of correlation and are therefore harder to recover. Moreover, it is well-established that expression or nonexpression of a particular gene may have distinct consequences in different tissues and disease states. Thus, our interpretation of the selected subset is that the selected genes are merely useful in discriminating between the 5 cancer types using gene expression patterns and are not necessarily linked to causal mechanisms. The sign and magnitude of each gene’s slope coefficient may aid practitioners in generating interesting hypotheses for follow-up studies. Incidentally, many of the selected genes are designated as “cancer enhanced” or “cancer enriched” by The Human Protein Atlas (Uhlen et al., 2017). For example:

- The transcription factor *HAND2*, which was selected in both breast (BRCA), colon (COAD), and lung (LUAD) cancers, is enriched in breast fibroblasts but not necessarily enriched in either cancer type.
- The transcription factor *CDX1*, selected in the colon adenocarcinoma cancer class (COAD), is expressed in the intestine and enriched in colorectal cancer.
- The gene product of *NKX2-1*, selected in the lung adenocarcinoma cancer class (LUAD), is expressed in lung tissue, for example alveolar cells, as well as thyroid gland tissue. It is enirched in both lung and thyroid cancers and specifically designated a cancer-related gene.
- In prostate adenocarcinoma (PRAD), the family of *KLK* genes *KLK2*, *KLK3*, and *KLK4* are enriched in both the prostate and prostate cancer. They are designated as cancer-related genes as it is highly expressed in prostate tumors, but it is not a prognostic marker.

### 4.4. Nonlinear classifier benchmarks

Next, we consider applications of sparse VDA to selecting the avatars in nonlinear, kernel-based VDA. Sparse models have fewer avatars. Table 6 summarizes our results. Nonlinear VDA is most successful in estimating a flexible decision boundary with few avatars in the simulated datasets **clouds**, **circles**, **spiral**, and **spiral-hard** datasets. The reported testing error interval for **circles** compares favorably to error rates previously reported by Wu and Wu (2012), even though it is larger than the irreducible error (20%) used in generating the data. The testing subset for dataset **vowel** is, by design, more difficult than the training data so we naturally expect a relatively higher classification error. Our improvement in median prediction error (5%) over the linear VDA classifiers in Table 5 (52%) on the **vowel** example is consistent with previous findings (Wu and Wu, 2012) for nonlinear VDA (VDA_K_). In each example, our sparse VDA classifiers select a few instances to serve as avatars driving a nonlinear decision boundary. Unlike the linear case, the use of class-specific avatar selection does not appear to improve nonlinear VDA prediction accuracy, as we attribute the improvement in the **vowel** dataset to tuning the kernel in nonlinear VDA.

### 4.5. Molecular subtypes in breast cancer

Molecular characterization of various cancers developed over the last two decades to better understand biological processes underlying disease and identify potential therapeutic targets (Denkert and Loibl, 2022). Subtyping of breast cancer is particularly notable as it evolved from classic immunohistochemistry markers to prognostic tools incorporating genetic signatures to predict recurrence risk. Modern efforts seek enhance molecular subtypes by linking gene expression profiles, immune cell infiltration, and other data to response outcomes (Denkert and Loibl, 2022; Wolf et al., 2022). Two important aspects are to address the existence of multiple classes and to identify small, *stable* subsets of features for downstream analyses robust to various batch effects (Szymiczek et al., 2021).

In this example we focus on predicting PAM50 molecular subtypes (Parker et al., 2009) for n=1089 breast cancer tumors in TCGA using p=35268 genes quantified via RNA sequencing. The dataset, TCGA-BRCA, recently received updates to normalize data and to include various histologic annotations which are of interest in understanding rarer forms of the disease (Thennavan et al., 2021). We focus on the molecular subtypes, Basal-like, Luminal A, Luminal B, HER2, and Normal-like, which in turn are not rigid classes but instead exhibit their own heterogeneity. Application of our sparse VDA methods to this example illustrates the utility of incorporating sparsity constraints into VDA for the purpose of extracting small gene subsets in the presence of ambiguity. Table 7 reports that our group-specific variable selection, HetL0, identifies a small, stable set of genes across 10 cross validation replicates. In comparison, the HetL1 VDA struggles to select sparse sets of genes in spite of its slight edge over L1R-SVM in terms of accuracy. It should also be noted that the dataset has gross class imbalance due to the ill-defined Normal-like class, which is represented by a mere 40 samples. Class imbalance likely factors into the stability of selection. Table 7 also indicates that class-heterogeneous sparsity recovers a far smaller, stable set compared to its homogeneous counterpart. Both methods are considerably more conservative compared to HetL1 VDA at the expense of some classification accuracy. It is unclear in this example whether either of the three VDA approaches have an obvious advantage. Indeed, the results for L1R-SVM suggest that careful tuning may allow the HetL1 VDA achieve a sparser subset via shrinkage. Interested readers may consult the appendix for notes on preprocessing using TCGABiolinks (Colaprico et al., 2016; Mounir et al., 2019) and a brief summary of overlap with the PAM50 gene set are available in Appendix D.

### 4.6. Ancestry-informative markers in human populations

Our final example applies sparse VDA to a subset of data from the 1000 Genomes Project (1kGP) (Genomes Project Consortium, 2015) for the purpose of extracting a sparse set of features predictive of human ancestry. The source data is based on the 2012 Omni Platform genotypes (pairs of alleles at various loci along DNA) restricted to unrelated human individuals (1,718 people) from 26 populations. Each included individual has a genotyping success rate of at least 95%. Data are further restricted to DNA sites with minor allele frequencies of at least 1%, resulting in 1,854,622 single nucleotide polymorphisms (SNPs) distributed across 22 autosomes to serve as features. The 1kGP represents an international effort to catalogue and analyze data on human variation for the purpose of understanding human genetic histories. In biomedical applications, adjusting for genetic ancestries is paramount as population stratification is known to induce false associations between SNPs, here treated as predictors, and medically-relevant traits, here treated as response variables (Li, 1969; Knowler et al., 1988; Marchini et al., 2004). Due to the size of the human genome, data on human variation at the DNA level are universally underdetermined. It therefore of interest to identify ancestry-informative markers (AIMs) (Shriver et al., 2003; Brown and Pasaniuc, 2014), small subsets of SNPs with strong correlations to ancestral groups, for the purpose of improving statistical estimation of admixture coefficients. A classical approach to ancestry adjustment involves extracting a few principal components of the SNP data to serve as predictors in an analysis. While successful, this approach is criticized for its weak interpretability and potential inefficienies in correcting bias (Lawson et al., 2020). Modern data mining techniques make it possible to extract AIMs to satisfy parsimony and interpretability requirements.

Our application of VDA to the 1kGP data focuses exclusively on Chromosome 1 in n=1,631 unrelated individuals and p=146,672 SNPs. Further, our analysis considers groupings of the 26 populations into 5 superpopulations: African, admixed American, East Asian, European, and South Asian. These superpopulations are not our own designations; they are available in the original data. Our demonstration is restricted to less than 10% of the original features. This is due to the SNP data being encoded as matrices with entries in {0, 1, 2}, understood as unsigned integers, for memory efficiency purposes. Linear Algebra Package (LAPACK) distributions do not provide subroutines for computing SVDs of such matrices, which are required by the present version of sparse VDA. Restricting our example to Chromosome 1 allows us to cast the data in double-precision floating point. Nevertheless, our results make a compelling case for the variable selection potential of sparsity constraints on data like the 1kGP; see for example a recent hard clustering approach due to Ko et al. (2023). Table 8 reports classification accuracy for HomL0, HetL0, and HetL1. The class-specific HetL0 VDA, which selects different subsets of variables for each class, has a slight edge in classification accuracy. However, as in Table 7, the size of the most stable subset of genes across cross validation replicates is considerably smaller for HetL0 compared to HomL0. This is likely an artifact due to our grid search heuristic which allows the features to vary across classes but imposes equal subset sizes in each group. Thus, while a greedy search based on explicit sparsity constraints may recover sparse models compared to shrinkage-based methods, this example demonstrates that the heuristic may introduce false positives in group variable selection.

## 5. Discussion and concluding remarks

The novelty of the current version of VDA stems from the application of 𝜖-insensitive loss, a new majorization, distance penalties, and projection onto sparsity sets. Sequential minimization via the surrogate function (4) reduces to least squares with shifted responses for fitting VDA classifiers.

Our work has its limitations. While our empirical results are satisfying, we have not characterized the statistical properties of our estimators. We lack conditions implying consistency of sparse VDA. Statistical theory is also needed to understand stability, or lack thereof, of our greedy variable selection procedure. Yet, Fig. 6 does suggest that VDA is strongly biased towards selecting sparse models on datasets with a small number of informative features. Unfortunately, cross validation replicates often yield candidate models with different numbers of active features. Dense models can be recovered, as illustrated by the prostate cancer example summarized in Fig. 6. In practice only one or just a few driving features may be selected out of a cluster of highly correlated features. This tendency may be beneficial in finding causal features in biological models. Finally, our code implementation relies heavily on BLAS calls to exploit parallelism in linear algebra operations. Additional parallel structure is potentially neglected.

We defer reporting timing benchmarks to Section C of the Supplementary Materials. Briefly, the most time consuming dataset to fit was TCGA-HiSeq, which required about 7 minutes per replicate on average. We emphasize that this feat involves solving thousands of nonconvex optimization problems as our algorithms (i) follow an annealing path to enforce sparsity via the strength of distance penalties, and (ii) search for an optimal model size. As sparse VDA moves along its solution path, it is only at the smallest model sizes, where classification is often nearly impossible, that our algorithms dramatically slow down. In addition, we note that the initial tuning phase is usually quite fast in practice. Our experience suggests that fitting sparse linear VDA models is much faster than fitting nonlinear VDA models. Whereas our proposed methods lack statistical guarantees, they are supported by theoretical guarantees on algorithmic convergence from the proximal disatance algorithms literature.

Repeated cross validation is not the only viable technique for evaluating candidate models. For instance, the method of *stability selection* described by Meinshausen and Bühlmann (2010) is attractive for its theoretical finite-sample guarantees in controlling family-wise errors. The *percentile lasso* is another attractive device for stabilizing model selection (Roberts and Nowak, 2014). The percentile lasso attempts to control variability by using the median of selected hyperparameter values. Both methods mentioned here, unfortunately, do not necessarily scale to truly high dimensional problems in which the number of covariates far exceeds the available number of samples. We fully recognize that our own sparse VDA classifiers are similarly limited in this regime because (a) the onerous demands of grid optimization to tune the optimal model size k are prohibitive without some prior information, and (b) the update step in our algorithms assumes the design matrix X is dense. Pioneering work on distance-to-set priors (Presman and Xu, 2023) may offer insights into incorporating Bayesian inference into proximal distance methods, thus addressing the former concern. *Screening techniques* that capitalize on recent advances in variable selection (Fan and Lv, 2008; Fan et al., 2009) can assist in reliably reducing the number of covariates in high dimensional datasets (Xie et al., 2020).

Finally, we draw the readers attention to how mixed integer programming can significantly improve the computational tractability of best subset selection in sparse regression (Bertsimas et al., 2016; Bertsimas and King, 2017; Bertsimas and Parys, 2020). Hastie et al. (2020) documented the beneficial effects of mixed integer programming in $\ell_{0}$-constrained least squares over penalized least squares, including robust nonconvex penalties such as SCAD (Fan and Li, 2001) and MCP (Zhang, 2010). Bertsimas et al. (2021) recently applied their discrete optimization techniques to sparse binary classification. At the time of our writing, the software provided by Bertsimas et al. (2021) only supports binary classification. Thus, we omit comparisons to this body of literature. However, our own experiments echo an important observation reported by Hastie et al. (2020) with respect to support recovery; namely, shrinkage-based estimators tend to perform relatively well in the low SNR regime while $\ell_{0}$-based methods often outperform the lasso in the high SNR regime.

In conclusion, we have demonstrated both the prediction accuracy and the feature selection capabilities of our sparse VDA algorithms across a range of simulated and real datasets. Our numerical examples confirm the promise of sparsity-based variable selection in VDA via distance penalties. Combining our estimation procedures with cross validation overcomes the need to specify a particular subset of variables. Replicating cross validation further improves the reliability of variable selection with sparse VDA and provides a straightforward mechanism for honest reporting. In the case of kernel VDA, our proposed methods allow for the simultaneous selection of the support vectors defining the decision boundaries separating multiple classes. We show that sparse VDA can recover a relatively stable set of features even in the underdetermined regime. In addition, we demonstrate the ease of designing penalties for class-specific variable selection and provide empirical evidence that our penalties can improve prediction and interpretation in classification. We hope to address gaps in theory and practice in future publications. In the meantime we hope readers will agree that sparse VDA clustering is ripe for further applications.

## Supplementary Material

supple

## Figures and Tables

**Table 1:** Summary of datasets and corresponding cross validation settings. Values in parentheses indicate the number of samples, features, or classes dropped from the original dataset.

	# classes	# samples	# features	# folds	Train / Test	References
leukemia	2	72	3571	3	58 / 14	Golub et al. (1999)
prostate	2	102	6033	3	82 / 20	Singh et al. (2002)
colon	2	62	2000	3	50 / 12	Alon et al. (1999)
SRBCT	4	63	2308	3	50 / 13	Khan et al. (2001)
lymphoma	3	62	4026	3	50 / 12	Pomeroy et al. (2002)
brain	5	42	5597	3	34 / 8	Alizadeh et al. (2000)
iris	3	150	4	3	120 / 30	Dua and Graff (2019)
lymphography	4	148	18	3	105 / 43	Dua and Graff (2019)
zoo	7	101	16 (1)	3	91 / 10	Dua and Graff (2019)
bcw	2	699	9	5	562 / 137	Dua and Graff (2019)
splice	3	3,186	180	5	2,549 / 637	Dua and Graff (2019)
letters	26	20,000	16	5	16,000 / 4000	Dua and Graff (2019)
optdigits	10	5,620	64	5	3,823 / 1797	Dua and Graff (2019)
vowel	11	990	10	5	528 / 462	Hastie et al. (2001)
HAR	6	10,299	561	5	7,352 / 2947	Dua and Graff (2019)
TCGA-HiSeq	5	801	20,264	4	601 / 200	Dua and Graff (2019)
BRCA	5	1,089	35,268	5	762 / 327	
1kGP	5	1,631	146,672	5	1,142 / 489	Genomes Project Consortium (2015)

**Table 2:** Timing and test error results for synthetic data with homogeneous classes, sumarized over 10 simulation replicates. The time column reflects costs due to cross validation and fitting the final model in each scenario, for each method. Here the number of predictors p=1000 and true predictors $k_{*}=30$ are fixed. The value n∈{500,2000} indicates the number of instances used to select and fit a candidate model. Test error is assessed using an additional 1000 independent samples. **Boldface** text is used to highlight the best results on the basis of median values.

				Time (s)				Test Error (%)			
*n*	*c*	*ρ*	SNR	HomL0	L1	MGSDA	L1R-SVM	HomL0	L1	MGSDA	L1R-SVM
500	3	0.1	0.1	3.61, 3.76, 4	**2.32, 2.44, 2.51**	3.85, 3.96, 4.06	8.21, 8.46, 9.57	**65, 66, 68**	**64, 66, 68**	**64, 66, 69**	**65, 66, 67**
500	3	0.5	0.1	3.66, 3.86, 3.95	**2.53, 2.59, 2.7**	3.69, 3.83, 3.99	7.66, 7.88, 8.11	57, 62, 62	**50, 53, 57**	51, 54, 56	**51, 53, 56**
500	3	0.9	0.1	3.82, 4.01, 4.06	**3.17, 3.22, 3.35**	5.41, 5.72, 5.88	7.35, 7.46, 7.67	33, 41, 45	22, 24, 27	**22, 22, 25**	23, 24, 26
500	3	0.1	1.0	3.49, 3.53, 3.58	**2.25, 2.29, 2.32**	3.67, 3.83, 3.91	8.14, 8.48, 9.57	**64, 65, 67**	65, 66, 67	64, 67, 68	64, 66, 68
500	3	0.5	1.0	3.66, 3.81, 4.06	**2.51, 2.55, 2.68**	3.79, 3.9, 4.07	7.97, 8.3, 8.52	56, 59, 63	50, 53, 55	**50, 52, 54**	52, 54, 56
500	3	0.9	1.0	4.15, 4.35, 4.56	**3.16, 3.23, 3.36**	4.98, 5.62, 5.82	7.45, 7.51, 7.69	**10, 14, 33**	21, 25, 28	20, 24, 26	21, 26, 28
500	3	0.1	10.0	3.42, 3.53, 3.56	**2.23, 2.27, 2.3**	3.7, 3.82, 3.96	8.16, 8.39, 8.86	61, 66, 67	64, 66, 68	**63, 65, 66**	**64, 65, 67**
500	3	0.5	10.0	3.57, 3.67, 3.75	**2.41, 2.44, 2.49**	3.86, 3.97, 4.12	8.04, 8.32, 8.45	27, 58, 63	52, 53, 55	**49, 52, 55**	50, 53, 56
500	3	0.9	10.0	3.76, 4.03, 4.17	**3.07, 3.15, 3.22**	5.2, 5.39, 5.64	7.28, 7.47, 7.6	**0, 0, 0.01**	21, 24, 26	22, 24, 26	24, 25, 26
500	10	0.1	0.1	5.26, 5.59, 6.07	4.77, 5.1, 5.32	**2.54, 2.74, 3.03**	49.3, 51, 55.4	88, 90, 91	**88, 89, 90**	88, 90, 90	**88, 89, 91**
500	10	0.5	0.1	5.79, 5.95, 6.07	5.72, 5.8, 5.89	**3.77, 4.05, 4.35**	47.2, 47.8, 48.3	84, 86, 87	**81, 84, 85**	75, 77, 79	**80, 84, 85**
500	10	0.9	0.1	**6.15, 6.53, 6.59**	7.62, 7.65, 7.82	17, 18.2, 19.2	40.7, 40.9, 41.1	67, 72, 75	53, 54, 58	**41, 43, 45**	51, 53, 57
500	10	0.1	1.0	5.22, 5.51, 5.81	4.75, 5, 5.07	**2.65, 2.98, 3.15**	49.5, 50.2, 56.5	**88, 89, 91**	88, 89, 90	**88, 89, 91**	87, 90, 90
500	10	0.5	1.0	5.9, 6.31, 6.87	5.45, 5.6, 5.76	**3.73, 4.02, 4.31**	48, 49.1, 50.3	84, 87, 88	81, 83, 85	**75, 77, 82**	82, 83, 86
500	10	0.9	1.0	**7.2, 7.41, 7.69**	7.64, 7.78, 7.99	18.1, 18.8, 19.6	41.2, 41.3, 41.7	**9.6, 14, 20**	55, 57, 58	41, 45, 47	55, 56, 58
500	10	0.1	10.0	5.09, 5.38, 5.89	4.81, 5.03, 5.08	**2.77, 2.95, 3.19**	49.9, 51.4, 56.2	90, 91, 91	89, 90, 91	**89, 89, 91**	89, 90, 91
500	10	0.5	10.0	5.48, 5.69, 5.9	5.49, 5.55, 5.59	**3.87, 4.17, 4.46**	48.9, 49.5, 52.6	84, 86, 87	81, 82, 84	**72, 77, 79**	82, 83, 85
500	10	0.9	10.0	**6.77, 6.98, 7.15**	7.61, 7.66, 7.73	18.5, 19.7, 20.8	40.7, 40.9, 41.2	**0, 0, 0.23**	54, 56, 58	42, 44, 47	54, 54, 56
2000	3	0.1	0.1	13.1, 13.5, 14.5	**8.16, 8.35, 8.79**	17.6, 18.3, 19.1	65, 75, 88.7	**46, 47, 49**	54, 57, 59	55, 57, 59	56, 58, 59
2000	3	0.5	0.1	15.4, 15.6, 15.9	**8.32, 8.54, 8.83**	17.4, 18.4, 19.1	154, 168, 176	**37, 39, 41**	40, 41, 43	41, 42, 43	41, 43, 44
2000	3	0.9	0.1	14.2, 14.7, 15.5	**8.34, 8.69, 9.36**	33.6, 34.6, 35.6	182, 192, 201	21, 24, 25	**16, 17, 19**	17, 18, 19	18, 18, 19
2000	3	0.1	1.0	13.3, 13.4, 14	**11.2, 11.7, 12.6**	53.6, 59.9, 70.9	214, 217, 223	**11, 12, 13**	17, 19, 20	19, 21, 22	20, 24, 24
2000	3	0.5	1.0	15.4, 15.9, 16.5	**11, 11.4, 11.6**	61, 65.9, 73.3	234, 240, 245	**10, 11, 13**	13, 16, 17	14, 16, 19	16, 19, 20
2000	3	0.9	1.0	17.8, 18.3, 19.6	**10.5, 10.8, 11.5**	65.6, 75.8, 81.4	182, 187, 189	7.5, 8, 9.1	6.4, 7.3, 8.1	**5.4, 6.4, 8**	7.1, 8.1, 9
2000	3	0.1	10.0	12, 12.7, 12.9	**9.96, 10.6, 11**	86.7, 93.6, 101	128, 142, 150	**0, 0, 0**	**0, 0, 0.1**	**0, 0, 0**	**0, 0, 0.11**
2000	3	0.5	10.0	13.4, 13.9, 14.4	**9.19, 9.82, 10.2**	92.4, 104, 124	150, 166, 181	**0, 0, 0**	**0, 0, 0.11**	**0, 0, 0**	**0, 0, 0.1**
2000	3	0.9	10.0	16.8, 17.5, 18.4	**9.88, 10.2, 11**	117, 126, 157	141, 154, 159	**0, 0, 0**	**0, 0, 0**	**0, 0, 0**	**0, 0, 0.2**
2000	10	0.1	0.1	22.6, 23.3, 24	**13.1, 13.6, 13.9**	21.2, 26.5, 31.2	194, 205, 214	**64, 66, 66**	80, 81, 83	82, 84, 84	80, 81, 83
2000	10	0.5	0.1	24.6, 25.3, 26.2	**13.3, 13.6, 14.4**	29.1, 32, 34.5	197, 203, 213	**56, 58, 60**	69, 71, 72	64, 66, 67	70, 73, 74
2000	10	0.9	0.1	23.9, 24.5, 25.5	**15.3, 15.3, 15.7**	69.7, 75, 77.8	302, 305, 308	42, 45, 50	34, 36, 40	**31, 33, 35**	36, 37, 41
2000	10	0.1	1.0	28.9, 29.3, 30.8	**17.3, 18, 18.7**	138, 153, 179	486, 496, 505	**5.9, 6.6, 7.5**	22, 25, 29	32, 35, 38	28, 31, 33
2000	10	0.5	1.0	31, 32, 32.7	**17.2, 18, 19.2**	174, 193, 207	462, 474, 477	**5.6, 6.5, 7.2**	18, 20, 23	21, 22, 25	24, 25, 27
2000	10	0.9	1.0	32.4, 33.6, 35.4	**18.7, 19.3, 20.5**	313, 333, 347	346, 347, 352	5, 5.6, 7.1	8.5, 9.9, 11	**4.4, 5.1, 5.7**	9.3, 10, 11
2000	10	0.1	10.0	25.4, 26.1, 26.9	**17.4, 18.1, 18.8**	434, 493, 541	460, 482, 504	**0, 0, 0.63**	**0, 0, 0**	**0, 0, 0**	**0, 0, 0**
2000	10	0.5	10.0	26.4, 27.6, 29	**17.6, 17.8, 18.8**	563, 581, 595	444, 453, 458	**0, 0, 7.8**	**0, 0, 0.01**	**0, 0, 0**	**0, 0, 0**
2000	10	0.9	10.0	29.5, 30.3, 30.7	**18.7, 19.4, 20**	846, 926, 998	337, 344, 350	**0, 0, 0**	**0, 0, 0.21**	**0, 0, 0**	**0, 0, 0.01**

**Table 3:** Timing and test error results for synthetic data with heterogeneous classes with p=1000 predictors. Each class is assigned a random subset of size $\left\lfloor 30/c\right\rfloor$ to act as true predictors. 10 simulation replicates are summarized with 10%, 50%, and 90% quantiles. **Boldface** text is used to highlight the best results on the basis of median values.

				Time (s)				Test Error (%)			
*n*	*c*	*ρ*	SNR	HomL0	HetL0	L1	HetL1	HomL0	HetL0	L1	HetL1
500	3	0.1	0.1	3.34, 3.43, 3.62	3.13, 3.22, 3.28	2.13, 2.22, 2.3	**2.04, 2.12, 2.2**	63, 65, 67	64, 65, 66	**64, 64, 68**	63, 65, 68
500	3	0.5	0.1	3.49, 3.83, 3.92	3.37, 3.5, 3.89	2.52, 2.59, 2.63	**2.38, 2.45, 2.51**	56, 59, 63	56, 58, 62	**52, 54, 55**	**52, 54, 57**
500	3	0.9	0.1	3.55, 3.63, 3.98	3.32, 3.46, 3.67	3.08, 3.13, 3.23	**2.92, 2.97, 3.03**	41, 45, 48	35, 44, 46	**23, 25, 27**	**22, 25, 27**
500	3	0.1	1.0	3.48, 3.58, 3.65	3.23, 3.3, 3.34	2.19, 2.23, 2.25	**2.07, 2.13, 2.15**	58, 64, 66	**52, 63, 66**	**60, 63, 66**	**60, 63, 65**
500	3	0.5	1.0	3.47, 3.71, 3.85	3.33, 3.37, 3.42	2.36, 2.4, 2.43	**2.26, 2.28, 2.33**	**26, 41, 56**	**25, 41, 56**	47, 51, 54	48, 51, 54
500	3	0.9	1.0	4.12, 4.18, 4.23	3.73, 3.79, 3.87	3.08, 3.14, 3.18	**2.93, 2.99, 3.01**	**11, 14, 21**	12, 16, 35	19, 21, 25	19, 21, 25
500	3	0.1	10.0	3.44, 3.61, 3.81	3.19, 3.33, 3.39	2.1, 2.21, 2.24	**2.04, 2.12, 2.19**	0.09, 61, 66	**0.19, 33, 65**	56, 64, 67	36, 61, 66
500	3	0.5	10.0	3.58, 3.78, 3.85	3.31, 3.36, 3.41	2.36, 2.38, 2.41	**2.23, 2.28, 2.33**	**0.18, 25, 59**	6.4, 29, 59	30, 41, 54	33, 43, 53
500	3	0.9	10.0	3.99, 4.1, 4.19	3.68, 3.74, 3.83	3.12, 3.2, 3.23	**2.95, 3.04, 3.16**	**0, 0, 0**	**0, 0, 0**	4, 8.7, 12	4.4, 7.7, 12
500	10	0.1	0.1	5.28, 5.47, 5.59	4.67, 4.8, 4.92	5.07, 5.21, 5.63	**4.29, 4.42, 4.76**	84, 86, 88	79, 80, 84	77, 79, 81	**76, 78, 81**
500	10	0.5	0.1	5.84, 5.99, 6.23	5.2, 5.24, 5.41	5.87, 5.95, 6.26	**5.05, 5.18, 5.37**	81, 82, 83	72, 75, 79	**63, 64, 66**	**63, 64, 66**
500	10	0.9	0.1	6.06, 6.14, 6.2	**5.73, 5.79, 5.83**	7.87, 7.9, 7.94	6.77, 6.82, 6.86	62, 69, 72	51, 56, 70	27, 29, 33	**26, 28, 31**
500	10	0.1	1.0	4.78, 4.87, 4.98	**3.41, 3.46, 3.51**	5.96, 6.03, 6.12	4.88, 4.94, 4.99	6.4, 15, 17	**5.4, 6.6, 7.7**	9.5, 9.9, 11	8.1, 9.5, 10
500	10	0.5	1.0	4.76, 4.84, 4.93	**3.39, 3.54, 3.66**	6.39, 6.48, 6.53	5.26, 5.28, 5.36	6.9, 8.5, 11	**5.3, 6.2, 7.2**	6.2, 8.1, 9.5	5.7, 8.2, 9.4
500	10	0.9	1.0	5.28, 5.32, 5.4	**4.15, 4.21, 4.29**	7.97, 8.03, 8.09	6.76, 6.83, 6.87	7.8, 8.7, 10	4.5, 6, 6.7	**1.8, 2.7, 3.3**	1.9, 2.8, 3.1
500	10	0.1	10.0	3.87, 3.98, 4.09	**2.07, 2.08, 2.13**	5.03, 5.16, 5.27	4.04, 4.16, 4.23	**0, 0, 0**	**0, 0, 0.19**	0, 0.05, 0.1	**0, 0, 0.11**
500	10	0.5	10.0	3.99, 4.03, 4.11	**2.2, 2.22, 2.26**	5.82, 5.94, 6.03	4.76, 4.83, 4.84	**0, 0, 0**	**0, 0, 0**	**0, 0, 0.01**	**0, 0, 0.1**
500	10	0.9	10.0	4.57, 4.6, 4.67	**2.85, 2.86, 2.88**	7.32, 7.46, 7.58	6.29, 6.35, 6.42	**0, 0, 0**	**0, 0, 0**	**0, 0, 0.12**	**0, 0, 0.2**
2000	3	0.1	0.1	14.1, 14.3, 14.6	13.5, 13.8, 14.2	8.29, 8.56, 9	**8.3, 8.51, 8.82**	**44, 48, 50**	47, 49, 51	52, 53, 55	52, 53, 55
2000	3	0.5	0.1	19.6, 20.8, 22.3	19, 20.4, 21.1	**10.3, 11, 11.8**	10.4, 11.4, 12.4	38, 40, 43	39, 41, 45	**37, 39, 41**	**37, 39, 41**
2000	3	0.9	0.1	13.8, 14, 14.2	13.5, 13.7, 13.9	8.45, 8.56, 8.64	**8.26, 8.34, 8.47**	22, 25, 30	22, 23, 25	**16, 17, 19**	**15, 17, 19**
2000	3	0.1	1.0	12.8, 13, 13.3	12.5, 12.8, 13.1	10.4, 10.7, 10.8	**10, 10.3, 10.6**	**11, 11, 13**	**11, 11, 14**	13, 14, 16	12, 14, 16
2000	3	0.5	1.0	13.7, 14.1, 14.2	13.4, 13.7, 14	10.2, 10.5, 10.8	**10.2, 10.2, 10.6**	**11, 12, 13**	**11, 12, 13**	12, 14, 15	12, 14, 15
2000	3	0.9	1.0	16, 16.4, 16.8	15.2, 15.5, 15.9	10.2, 10.4, 10.4	**9.99, 10.3, 10.5**	6.8, 7.7, 9.5	**6.1, 7.5, 8.4**	4.9, 5.9, 6.3	5, 5.8, 6.7
2000	3	0.1	10.0	12.8, 13, 13.1	12.5, 12.7, 13	7.61, 7.89, 8.12	**7.37, 7.57, 7.78**	**0, 0, 0.1**	**0, 0, 0**	**0, 0, 0**	**0, 0, 0**
2000	3	0.5	10.0	13.5, 13.8, 14.6	13.6, 14, 14.6	9.19, 9.5, 9.74	**8.95, 9.19, 9.31**	**0, 0, 0**	**0, 0, 0**	**0, 0, 0**	**0, 0, 0**
2000	3	0.9	10.0	16.1, 16.5, 16.9	16.3, 16.4, 16.7	10.4, 10.8, 11.1	**10.3, 10.6, 10.8**	**0, 0, 0**	**0, 0, 0**	**0, 0, 0.1**	**0, 0, 0.01**
2000	10	0.1	0.1	21.2, 22.1, 23.5	19.8, 20.9, 23.1	14, 14.5, 15.1	**13.2, 13.7, 14.7**	64, 65, 67	**62, 64, 66**	**63, 64, 67**	**62, 64, 66**
2000	10	0.5	0.1	24.3, 25.4, 26.2	23.9, 24.7, 26	15.4, 16.3, 17.5	**14.7, 15.4, 16.2**	57, 58, 59	50, 52, 53	**48, 49, 51**	**48, 49, 51**
2000	10	0.9	0.1	21.1, 21.2, 21.8	21.2, 21.4, 21.8	14.8, 15, 15.1	**14.2, 14.3, 14.4**	46, 52, 54	28, 30, 37	20, 22, 23	**20, 21, 23**
2000	10	0.1	1.0	23.5, 24, 24.5	22, 22.1, 22.7	11.5, 11.5, 11.7	**10.6, 10.7, 10.9**	5.9, 6.2, 7.2	**5.4, 5.7, 6.4**	5.3, 5.8, 7.1	4.7, 6, 6.9
2000	10	0.5	1.0	23.4, 23.8, 24.1	21.6, 21.9, 22	15.9, 16.4, 16.5	**14.9, 15.2, 15.5**	5.6, 6.6, 7	4.8, 6, 6.6	4.2, 4.5, 5.3	**4.2, 4.4, 5.5**
2000	10	0.9	1.0	24.5, 24.9, 25.2	22.1, 22.4, 22.8	20.3, 20.4, 20.6	**19.1, 19.2, 19.5**	4.9, 5.9, 6.5	3.9, 4.6, 5.1	**1.1, 1.6, 2.1**	1.2, 1.7, 2
2000	10	0.1	10.0	20.6, 21, 21.3	15.1, 15.1, 15.3	8.93, 9.12, 9.29	**8.23, 8.3, 8.43**	**0, 0, 0**	**0, 0, 0**	**0, 0, 0**	**0, 0, 0**
2000	10	0.5	10.0	21.7, 22, 22.5	14.7, 14.8, 15	12.5, 12.8, 13.2	**11.5, 11.8, 12.1**	**0, 0, 0**	**0, 0, 0**	**0, 0, 0**	**0, 0, 0**
2000	10	0.9	10.0	20.8, 20.9, 21.3	**13, 13.1, 13.4**	17.9, 18.1, 18.2	16.7, 16.8, 16.9	**0, 0, 0**	**0, 0, 0**	**0, 0, 0**	**0, 0, 0.01**

**Table 4:** Cancer benchmarks summarized by 10%, 50%, and 90% quantiles, based on 50 cross validation replicates. Results for VDA_LE_ are taken from Wu and Lange (2010); see Table 5. Classification errors for VDA_LE_ report standard errors rather than intervals. **Boldface** text highlights the best results in each example based on median values.

	VDA_LE_		HomL0		HetL0		HetL1	
	Error (%)	# Active	Error (%)	# Active	Error (%)	# Active	Error (%)	# Active
colon	**9.68 (0.55)**	10, 27, 103	8.3, 17, 34	18, 63, 341	0, 17, 33	20, 83, 413	7.5, 17, 33	74, 195, 909
SRBCT	1.58 (0.77)	45, 60, 94	**0, 0, 23**	15, 25, 72	**0, 0, 0.77**	20, 33, 68	**0, 0, 7.7**	74, 134, 244
leukemia	1.56 (0.15)	18, 39, 74	**0, 0, 7.9**	20, 58, 445	0, 7.1, 14	16, 40, 417	0, 3.6, 7.9	115, 278, 1408
lymphoma	1.66 (0.27)	39, 69, 97	0, 8.3, 25	28, 40, 187	**0, 0, 8.3**	51, 82, 326	**0, 0, 8.3**	557, 788, 1914
brain	23.8 (1.54)	52, 78, 98	0, 25, 38	70, 220, 1055	0, 19, 38	141, 372, 1426	**0, 12, 38**	912, 1962, 5387
prostate	**5.48 (0.33)**	16, 40, 53	0, 7.5, 15	18, 99, 730	4.5, 10, 20	40, 246, 940	0, 10, 15	49, 242, 1066

**Table 5:** Linear VDA benchmarks. Replicates are summarized as 10%, 50%, and 90% quantiles. Two examples are omitted for L1R-SVM due to long-running computation.

	HomL0		HetL0		HetL1		L1R-SVM	
	Error (%)	# Active	Error (%)	# Active	Error (%)	# Active	Error (%)	# Active
iris	0, 3.3, 10	2, 2, 3	0, 3.3, 7	3, 4, 4	0, 3.3, 10	4, 4, 4	10, 17, 23	3, 4, 4
lymphography	14, 21, 30	14, 28, 38	9.3, 21, 28	20, 38, 40	12, 19, 26	18, 41, 41	12, 19, 26	22, 30, 39
zoo	0, 10, 20	7, 12, 15	0, 10, 20	9, 14, 16	0, 0, 10	16, 16, 16	0, 10, 20	15, 16, 16
bcw	2.2, 3.6, 5.9	4, 7, 9	2.2, 3.6, 5.9	4, 8, 9	1.5, 3.3, 5.8	9, 9, 9	1.5, 2.9, 5.1	6, 8, 9
waveform	17, 19, 23	7, 12, 19	17, 19, 22	10, 16, 20	16, 17, 19	12, 16, 21	16, 18, 20	13, 16, 19
splice	4.5, 5.4, 6.1	43, 68, 124	4.5, 5.2, 6	64, 91, 138	4.2, 4.6, 5.4	148, 196, 213	8.9, 10, 11	165, 228, 239
letters	34, 35, 35	16, 16, 16	34, 35, 35	16, 16, 16	34, 35, 35	16, 16, 16	44, 45, 46	16, 16, 16
optdigits	3.7, 4.3, 5.2	55, 57, 60	3.7, 4.3, 4.9	62, 62, 62	3.6, 4.2, 4.8	59, 62, 64	7.6, 8.2, 8.9	62, 62, 62
vowel	50, 52, 56	6, 9, 10	50, 53, 56	10, 10, 10	49, 51, 54	10, 10, 10	55, 59, 62	10, 10, 10
HAR	1.4, 1.7, 2	220, 262, 321	1.5, 1.8, 2.1	285, 404, 504	1.5, 1.7, 2	561, 561, 561	Omitted	Omitted
TCGA-HiSeq	0, 0, 16	57, 326, 654	0, 0, 0.5	265, 516, 2336	0, 0, 0.55	4170, 6449, 15753	Omitted	Omitted

**Table 6:** Nonlinear VDA benchmarks. Replicates are summarized by 10%, 50%, and 90% quantiles. Results for VDA_K_ are taken from Wu and Wu (2012), which simulates the **clouds** example with a different value of σ.

	VDA_K_		HomL0		HetL0		HetL1	
	Error (%)	# Active	Error (%)	# Active	Error (%)	# Active	Error (%)	# Active
circles	**28.1 (2.4)**		26, 30, 34	109, 144, 200	26, 30, 34	188, 228, 250	26, 30, 34	248, 250, 250
clouds	10.6 (0.5)	∗𝜎 = 0.3	5, 36, 67	4, 54, 153	4.9, 34, 67	26, 93, 211	**4.5, 6.7, 67**	69, 227, 250
waveform	**14.5 (0.7)**		15, 17, 21	14, 39, 168	16, 19, 22	35, 74, 220	14, 16, 18	25, 74, 244
spiral			1.8, 15, 31	52, 178, 344	1.8, 15, 24	78, 245, 422	**1, 4.8, 22**	351, 500, 500
spiral-hard			20, 23, 52	136, 271, 334	20, 22, 31	269, 350, 423	**18, 21, 25**	347, 500, 500
Vowel	41.8		4.1, 5.4, 8.7	203, 276, 333	3.2, 5.2, 8.2	235, 332, 480	**3, 5, 7**	526, 528, 528

**Table 7:** Variable selection on TCGA-BRCA with n=1089 and p=35268. 10 cross validation replicates are summarized by 10%, 50%, and 90% quantiles. The column ‘# Active’ denotes the number of active genes across all classes. The column ‘Replicates, # Active’ reports the number of times the most stable subset size is replicated along with the subset size.

	HomL0		HetL0		HetL1		L1R-SVM	
	Error (%)	# Active	Error (%)	# Active	Error (%)	# Active	Error (%)	# Active
Total	13, 15, 16	3870, 8736, 30129	11, 14, 18	4817, 14676, 21043	13, 13, 16	35204, 35212, 35224	14, 17, 20	169, 318, 3510
	Error (%)	Replicates, # Active	Error (%)	Replicates, # Active	Error (%)	Replicates, # Active	Error (%)	

Basal	0, 1.9, 5.5	10, 804	0, 1.8, 5.4	10, 226	0, 1.7, 3.6	10, 35026	1.7, 3, 4.4	
Her2	23, 27, 38	10, 804	19, 23, 33	10, 166	16, 20, 23	10, 32532	13, 16, 20	
LumA	3.7, 5.3, 6.7	10, 804	3.3, 5, 7.5	10, 123	2.7, 5.3, 7	10, 2222	13, 16, 25	
LumB	30, 35, 42	10, 804	26, 35, 39	10, 141	29, 35, 45	10, 3263	0, 9, 41	
Normal	60, 78, 100	10, 804	66, 88, 95	10, 116	57, 85, 94	10, 33356	79, 91, 100	

**Table 8:** Variable selection on Chromosome 1 of the 1kGP data with n=1,631 individuals and p=146,672 SNPs. 10 cross validation replicates are summarized by 10%, 50%, and 90% quantiles. The column ‘# Active’ denotes the number of active genes across all classes. The column ‘Replicates, # Active’ reports the number of times the most stable subset size is replicated along with the subset size.

	HomL0		HetL0		HetL1	
	Error (%)	# Active	Error (%)	# Active	Error (%)	# Active
Total	1.9, 2.8, 8	2221, 8342, 18074	1.4, 2.2, 3.3	3076, 10304, 60641	2.2, 3.5, 3.8	142176, 146672, 146672
	Error (%)	Replicates, # Active	Error (%)	Replicates, # Active	Error (%)	Replicates, # Active

Admixed American	9.2, 14, 16	10, 338	7.2, 11, 16	10, 93	12, 20, 23	10, 2493
African	0, 2.3, 31	10, 338	0, 0.98, 2.9	10, 98	0, 0, 1.2	10, 66177
East Asian	0, 0, 0	10, 338	0, 0, 0	10, 297	0, 0, 0	10, 87982
European	0, 0, 0	10, 338	0, 0, 0.96	10, 50	0, 0, 0	10, 3186
South Asian	0, 0, 8.5	10, 338	0, 0, 0	10, 292	0, 0, 0	10, 29565
