# Supplementary material for: Sparse vertex discriminant analysis: Variable selection for biomedical classification applications
Source: Comput Stat Data Anal. Author manuscript; Available in PMC 2025 Jun 1. (PMC12122019; doi:10.1016/j.csda.2025.108125)
Supplement: supple [file NIHMS2073163-supplement-supple.pdf]

A Derivations

A.1 Directly minimizing surrogate (4)

According to the orthogonality relation $\boldsymbol{V}^\top \boldsymbol{V} = \boldsymbol{I}_r$ and Woodbury's formula, the required inverse is

$$\begin{aligned}
[n^{-1}\boldsymbol{V}\boldsymbol{\Sigma}^2\boldsymbol{V}^\top + c\boldsymbol{I}_p]^{-1} &= c^{-1}\left[\boldsymbol{I}_p - c^{-1}\boldsymbol{V}(n\boldsymbol{\Sigma}^{-2} + c^{-1}\boldsymbol{V}^\top\boldsymbol{V})^{-1}\boldsymbol{V}^\top\right] \\
&= c^{-1}\left[\boldsymbol{I}_p - c^{-1}\boldsymbol{V}(n\boldsymbol{\Sigma}^{-2} + c^{-1}\boldsymbol{I}_r)^{-1}\boldsymbol{V}^\top\right] \\
&= c^{-1}\left[\boldsymbol{I}_p - n^{-1}\boldsymbol{V}(c\boldsymbol{I} + n^{-1}\boldsymbol{\Sigma}^2)^{-1}\boldsymbol{\Sigma}^2\boldsymbol{V}^\top\right] \\
&= c^{-1}\left[\boldsymbol{I}_p - \boldsymbol{V}\Psi(c)\boldsymbol{V}^\top\right],
\end{aligned}$$

where the diagonal matrix $\boldsymbol{\Psi}(c) = n^{-1}\boldsymbol{\Sigma}^2(n^{-1}\boldsymbol{\Sigma}^2 + c\boldsymbol{I})^{-1}$ collects rescaled square singular values. Hence, the update (11) reduces to matrix-matrix multiplication

$$\boldsymbol{B}_{m+1} = \rho^{-1}\left[\boldsymbol{I}_p - \boldsymbol{V}\boldsymbol{\Psi}(\rho)\boldsymbol{V}^\top\right]\left(n^{-1}\boldsymbol{X}^\top\boldsymbol{Z}_m + \rho\boldsymbol{P}_m\right).$$

B Dataset descriptions and provenance

B.1 Preprocessing

Throughout each example we standardize each feature vector $\boldsymbol{x}_i$ to have mean $\boldsymbol{0}$ and unit variance by estimating sample means $\boldsymbol{\mu} = \frac{1}{n}\sum_{i=1}^n \boldsymbol{x}_i$ and variances $\boldsymbol{\sigma}^2 = \frac{1}{n-1}\sum_{i=1}^n(\boldsymbol{x}_i - \boldsymbol{\mu})^2$, where operations are applied element-wise. We then apply a Z-score transformation $\boldsymbol{x} \mapsto (\boldsymbol{x} - \boldsymbol{\mu})/\boldsymbol{\sigma}$. Location and scale estimates are based on a *training* subset only, and the Z-score transformation is applied to all subsets in cross validation.

For real-world datasets we take care to drop missing data. In addition, we make the choice to drop any non-numeric features (e.g. categorical data) *that are not already coded*. We note this in each description where applicable.

B.2 Gaussian clouds (clouds)

As described by Wu and Wu (2012), we simulate six Gaussian clouds with centers (x, y) equally spaced along the unit circle in $\mathbb{R}^2$.

$$\theta = \begin{cases} 0 \text{ or } \pi & \text{for class } 1, \\ \frac{\pi}{3} \text{ or } \frac{4\pi}{3} & \text{for class } 2, \\ \frac{2\pi}{3} \text{ or } \frac{5\pi}{3} & \text{for class } 3, \end{cases} \quad \text{with} \quad (x,y) = (\cos(\theta), \sin(\theta)).$$

Each class has equal probability and centers are sampled uniformly conditional on the chosen class. Points about a given cloud are simulated with variance-covariance matrix $\sigma^2\boldsymbol{I}_{2\times 2}$. Thus, higher variability increases class overlap and hence classification difficulty. We set $\sigma = 0.25$ and split the data into training, validation, and testing subsets of sizes 200, 50, and 1000, respectively, in 5-fold cross validation.

B.3 Nested circles (circles)

The recipe described by Wu and Wu (2012) samples two features from the circle $\{(x, y) : x^2 + y^2 < c\}$ and assigns them to one of c classes. The intermediate values $r = \lceil x^2 + y^2 \rceil$ and r' are used to simulate class assignment via the rule

$$\text{class} = \begin{cases} r & \text{with probability } p \\ r' & \text{with probability } 1 - p. \end{cases}$$

Here $\lceil\cdot\rceil$ is the ceiling function, r' is any class in $\{1, 2, \ldots, c\}$ other than r which is sampled uniformly, and $1 - p$ is the Bayes error controlling the difficulty of classification. Our benchmark takes $c = 3$ and $p = 0.8$ to simulate a dataset with 1250 samples. We split the data into training, validation, and testing subsets of sizes 200, 50, and 1000, respectively, in 5-fold cross validation.

B.4 Waveform

This dataset is based on convex combinations of triangular waveforms that was originally presented by Breiman et al. (1984) and featured in Example 12.7.1 of *The Elements of Statistical Learning* (Hastie et al., 2001). We simulate $p = 21$ features across $c = 3$ classes using the recipe

$$\begin{aligned} X_j &= U h_1(j) + (1-U) h_2(j) + \epsilon_j && \text{for class 1,} \\ X_j &= U h_1(j) + (1-U) h_3(j) + \epsilon_j && \text{for class 2,} \qquad j = 1, 2, \ldots, 21 \\ X_j &= U h_2(j) + (1-U) h_3(j) + \epsilon_j && \text{for class 3,} \end{aligned}$$

where U is a uniform deviate on $[0, 1]$ and the ϵ_j are independent standard normal deviates. The triangular waveforms $h_1(j) = \max\{6 - |j - 11|, 0\}$, $h_2(j) = h_1(j - 4)$, and $h_3(j) = h_1(j + 4)$ are centered at $j = 11$, $j = 15$, and $j = 7$, respectively. A total of 1375 samples are simulated with training, validation, and testing subsets of sizes 300, 75, and 1000, respectively, in 5-fold cross validation.

B.5 UCI datasets

We select 8 datasets from the UCI Machine Learning Repository (Dua and Graff, 2019), all of which are in the overdetermined regime. Table 1 summarizes the characteristics of each selected example.

Table 1: Summary of datasets from UCI Machine Learning Repository and corresponding cross validation settings. Values in parentheses indicate the number of samples, features, or classes dropped from the original dataset.

	# classes	# samples	# features	# folds	Train / Test
iris	3	150	4	3	120 / 30
lymphography	4	148	18	3	105 / 43
zoo	7	101	16 (1)	3	91 / 10
bcw	2	699	9	5	562 / 137
splice	3	3186	180	5	2549 / 637
letters	26	20000	16	5	16000 / 4000
optdigits	10	5620	64	5	3823 / 1797
HAR	6	10299	561	5	7352 / 2947

B.6 Cancer datasets

Our experiments on cancer microarray expression data is based on 6 preprocessed datasets as described by Dettling and Bühlmann (2002) and previously available from https://stat.ethz.ch/∼dettling/supercluster.html. The detailed study of Dettling and Bühlmann (2002) reports leave-one-out cross validation error rates on leukemia (1.39%), prostate cancer (4.90%), colon cancer (16.13%), Small-Blue-Round-Cell-Tumor (SBRCT) cancer (0.00%), lymphoma (0.00%), and brain cancer (11.90%) using a supervised clustering method that compared favorably against existing literature at the time. Notably, all 6 examples are in the underdetermined regime.

Table 2: Summary of cancer microarray expression data and corresponding cross validation settings.

	# classes	# samples	# features	# folds	Train / Test	References
leukemia	2	72	3571	3	58 / 14	Golub et al. (1999)
prostate	2	102	6033	3	82 / 20	Singh et al. (2002)
colon	2	62	2000	3	50 / 12	Alon et al. (1999)
SRBCT	4	63	2308	3	50 / 13	Khan et al. (2001)
lymphoma	3	62	4026	3	50 / 12	Pomeroy et al. (2002)
brain	5	42	5597	3	34 / 8	Alizadeh et al. (2000)

B.7 Vowel dataset

This dataset is taken from *Elements of Statistical Learning*. It consists of $n = 990$ samples with $p = 10$ features and $c = 11$ classes. We use the original train / test split of 528 / 462 in 5-fold cross validation. A full original description of the dataset is available at `https://hastie.su.domains/ElemStatLearn/`.

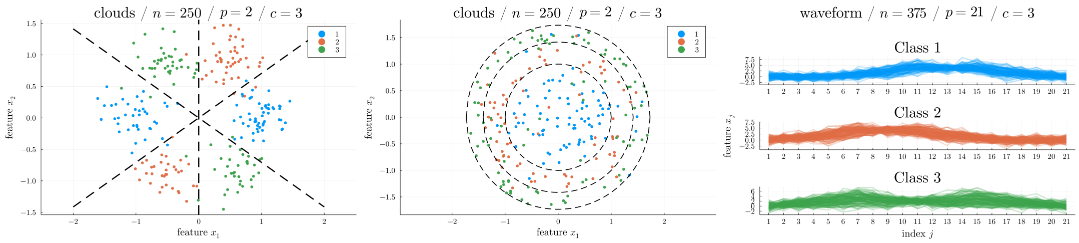

Figure 1: Representative subsets used in 5-fold cross validation for each simulated example. Here n, p, and c correspond to numbers of samples, features, and classes, respectively.

B.8 Visualizations of simulated examples

Figure 1 illustrates the shape of our simulated examples.

C Additional benchmarks

This section records aggregate timing results for various sparse VDA algorithms, which includes time taken to tune hyperparameters, perform variable selection, and fit a final candidate model. Results are based on multiple replicates of cross validation.

Table 3: Timing results for cancer microarray expression data sets, measured in seconds and reported as 10%, 50%, and 90% quantiles. Results are based on 50 replicates.

	HomL0	HetL0	HetL1
colon	1.17, 1.2, 1.23	1.14, 1.18, 1.22	2.74, 3.16, 3.47
SRBCT	0.903, 0.919, 0.997	1.1, 1.12, 1.23	3.01, 3.1, 3.24
leukemia	1.66, 1.71, 1.81	1.62, 1.65, 1.7	2.8, 2.83, 2.87
lymphoma	2.52, 2.57, 2.7	2.75, 2.84, 2.92	4.15, 4.17, 4.31
brain	5.68, 5.81, 6.05	7.77, 7.98, 8.17	9.26, 9.32, 9.48
prostate	4.84, 5.04, 5.11	4.93, 4.97, 5.12	5.31, 5.39, 5.53

Table 4: Timing results for linear VDA examples, measured in seconds and reported as 10%, 50%, and 90% quantiles. Results are based on 50 replicates except for those marked with an asterisk ($*$),which are based on 10 replicates.

	HomL0	HetL0	HetL1
iris	0.0115, 0.0118, 0.0122	0.0114, 0.0118, 0.0122	0.0126, 0.0131, 0.0137
lymphography	0.0992, 0.156, 0.202	0.102, 0.156, 0.188	0.0385, 0.0471, 0.0585
zoo	0.0424, 0.046, 0.101	0.0282, 0.0303, 0.0894	0.0276, 0.0305, 0.0487
bcw	0.169, 0.188, 0.212	0.169, 0.187, 0.208	0.208, 0.221, 0.243
waveform	0.106, 0.11, 0.132	0.0922, 0.0974, 0.11	0.0696, 0.0746, 0.0948
splice	6.16, 6.29, 6.48	5.4, 5.49, 5.64	2.24, 2.28, 2.33
letters	33.1, 33.8, 34.2	25.9, 26.4, 26.9	25.2, 25.6, 26
optdigits	9.83, 10, 10.2	9.24, 9.44, 9.73	5.78, 5.88, 5.97
vowel	0.225, 0.235, 0.243	0.211, 0.215, 0.224	0.181, 0.188, 0.197
HAR	37.9, 39.6, 64.4	29.4, 31.2, 59.7	39.9, 40.4, 42.1
TCGA-HiSeq*	410, 411, 416	428, 437, 468	197, 198, 200
BRCA*	589, 597, 615	609, 620, 642	550, 559, 562
TGP*	1900, 1910, 1990	2000, 2020, 2060	2920, 2950, 2960

D Variable selection in TCGA-BRCA and PAM50

Table 5 reports genes selected by our sparse VDA algorithms within the TCGA-BRCA dataset. Specifically, we report genes overlapping with the PAM50 gene set (Parker et al., 2009). The data were accessed using the TCGABiolinks software. For preprocessing, we filtered out duplicates and samples lacking molecular subtype annotations resulting

in $n = 1089$ samples. We adjusted raw counts ("unstranded" assay) using the `TCGAanalyze_Preprocessing` and `TCGAanalyze_Normalization` functions, leaving $p = 35,268$ genes for our application.

Table 5: Comparison of genes selected in TCGA-BRCA with PAM50 genes, over 10 cross validation replicates. Only genes selected in all 10 replicates are included. Cells prefixed with "+" indicate genes overlapping with PAM50. Cells prefixed with "-" indicate genes *missing* from the overlap with PAM50 (set difference). For example, "-:ORC6L" indicates all 50 genes were selected *except* ORC6L.

Method	Basal	Her2	LumA	LumB	Normal
HomL0	+:KRT5, KRT17, FOXC1, SFRP1, ESR1, GRB7, ERBB2	+:KRT5, KRT17, FOXC1, SFRP1, ESR1, GRB7, ERBB2	+:KRT5, KRT17, FOXC1, SFRP1, ESR1, GRB7, ERBB2	+:KRT5, KRT17, FOXC1, SFRP1, ESR1, GRB7, ERBB2	+:KRT5, KRT17, FOXC1, SFRP1, ESR1, GRB7, ERBB2
HetL0	+:FOXC1, MLPH, FOXA1, GPR160, TMEM45B	+:ESR1, GRB7, ERBB2		+:KRT17	
HetL1	-:ORC6L	-:UBE2T, ORC6L	+:NUF2, CEP55, CDC20, KRT5, PHGDH, KRT17, FOXC1, KRT14, MAPT, BCL2, NAT1, GRB7	+:EGFR, KRT5, CDH3, KRT17, FOXC1, SFRP1, KRT14, ESR1, GRB7, ERBB2	-:TYMS, ORC6L, MKI67, MAPT

E Additional details

This section addresses additional details not covered in the preceding appendices.

E.1 Class vertices and maximal dead zone ϵ

Encoding c classes into the standard simplex in R^c amounts to assigning each class to a standard basis vector $\boldsymbol{e}_j$ for $j = 1, 2, \ldots, c$. In this setting $\|\boldsymbol{e}_j - \boldsymbol{e}_i\| = \sqrt{2}$ for each nontrivial pair (i, j). Thus, the maximal value of ϵ that avoids overlapping dead zones is $\epsilon = 1/\sqrt{2}$, regardless of the number of classes c.

E.2 Computing environment

All numerical examples are run on a Linux desktop (Manjaro) equipped with 32 GB RAM and an Intel 10900KF processor locked at 4.9 GHz (10 cores). Our algorithms are implemented in the Julia language, version 1.7.3.

References

Alizadeh, A. A., M. B. Eisen, R. E. Davis, C. Ma, I. S. Lossos, A. Rosenwald, J. C. Boldrick, H. Sabet, T. Tran, X. Yu, J. I. Powell, L. Yang, G. E. Marti, T. Moore, J. Hudson, L. Lu, D. B. Lewis, R. Tibshirani, G. Sherlock, W. C. Chan, T. C. Greiner, D. D. Weisenburger, J. O. Armitage, R. Warnke, R. Levy, W. Wilson, M. R. Grever, J. C. Byrd, D. Botstein, P. O. Brown, and L. M. Staudt (2000). Distinct types of diffuse large B-cell lymphoma identified by gene expression profiling. *Nature 403*(6769), 503–511.

Alon, U., N. Barkai, D. A. Notterman, K. Gish, S. Ybarra, D. Mack, and A. J. Levine (1999). Broad patterns of gene expression revealed by clustering analysis of tumor and normal colon tissues probed by oligonucleotide arrays. *Proceedings of the National Academy of Sciences 96*(12), 6745–6750.

Breiman, L., J. Friedman, C. J. Stone, and R. A. Olshen (1984). *Classification and Regression Trees*. CRC press.

Dettling, M. and P. Bühlmann (2002). Supervised clustering of genes. *Genome Biology 3*(12), research0069.1.

Dua, D. and C. Graff (2019). UCI Machine Learning Repository.

Golub, T. R., D. K. Slonim, P. Tamayo, C. Huard, M. Gaasenbeek, J. P. Mesirov, H. Coller, M. L. Loh, J. R. Downing, M. A. Caligiuri, C. D. Bloomfield, and E. S. Lander (1999). Molecular Classification of Cancer: Class Discovery and Class Prediction by Gene Expression Monitoring. *Science 286*(5439), 531–537.

Hastie, T., J. Friedman, and R. Tibshirani (2001). *The Elements of Statistical Learning*. Springer Series in Statistics. Springer New York.

Khan, J., J. S. Wei, M. Ringnér, L. H. Saal, M. Ladanyi, F. Westermann, F. Berthold, M. Schwab, C. R. Antonescu, C. Peterson, and P. S. Meltzer (2001). Classification and diagnostic prediction of cancers using gene expression profiling and artificial neural networks. *Nature Medicine 7*(6), 673–679.

Parker, J. S., M. Mullins, M. C. Cheang, S. Leung, D. Voduc, T. Vickery, S. Davies, C. Fauron, X. He, Z. Hu, J. F. Quackenbush, I. J. Stijleman, J. Palazzo, J. Marron, A. B. Nobel, E. Mardis, T. O. Nielsen, M. J. Ellis, C. M. Perou, and P. S. Bernard (2009). Supervised Risk Predictor of Breast Cancer Based on Intrinsic Subtypes. *Journal of Clinical Oncology 27*(8), 1160–1167.

Pomeroy, S. L., P. Tamayo, M. Gaasenbeek, L. M. Sturla, M. Angelo, M. E. McLaughlin, J. Y. H. Kim, L. C. Goumnerova, P. M. Black, C. Lau, J. C. Allen, D. Zagzag, J. M. Olson, T. Curran, C. Wetmore, J. A. Biegel, T. Poggio, S. Mukherjee, R. Rifkin, A. Califano, G. Stolovitzky, D. N. Louis, J. P. Mesirov, E. S. Lander, and T. R. Golub (2002). Prediction of central nervous system embryonal tumour outcome based on gene expression. *Nature 415*(6870), 436–442.

Singh, D., P. G. Febbo, K. Ross, D. G. Jackson, J. Manola, C. Ladd, P. Tamayo, A. A. Renshaw, A. V. D'Amico, J. P. Richie, E. S. Lander, M. Loda, P. W. Kantoff, T. R. Golub, and W. R. Sellers (2002). Gene expression correlates of clinical prostate cancer behavior. *Cancer Cell 1*(2), 203–209.

Wu, T. T. and Y. Wu (2012). Nonlinear vertex discriminant analysis with reproducing kernels. *Statistical Analysis and Data Mining: The ASA Data Science Journal 5*(2), 167–176.
